# Investigating Aboriginal and Torres Strait Islander male health and wellbeing programs and their key elements: A scoping review

**DOI:** 10.1002/hpja.940

**Published:** 2024-12-09

**Authors:** Kootsy Canuto, Celina Gaweda, Bryce Brickley, Rosie Neate, Courtney Hammond, Leah Newcombe, Graham Gee, Oliver Black, Douglas Clinch, James A. Smith, Karla J. Canuto

**Affiliations:** ^1^ Flinders University, College of Medicine and Public Health, Flinders Health and Medical Research Institute Darwin Northwest Territories Australia; ^2^ Aboriginal Health Equity South Australian Health and Medical Research Institute Adelaide South Australia Australia; ^3^ Adelaide Medical School University of Adelaide Adelaide South Australia Australia; ^4^ Queensland Department of Education Cairns Queensland Australia; ^5^ Murdoch Children's Research Institute, Royal Children's Hospital Melbourne Melbourne Victoria Australia; ^6^ National Centre for Epidemiology and Population Health College of Health & Medicine, The Australian National University Acton Australian Capital Territory Australia

**Keywords:** first nations, health, indigenous, men's health, primary health care, quality improvement

## Abstract

**Issue Addressed:**

This scoping review aims to explore the size and scope of the body of literature relating to Aboriginal and Torres Strait Islander male health and wellbeing programs and describe key program elements.

**Methods:**

This review considered unpublished and published literature from electronic peer‐reviewed databases and grey literature sources. Included articles must refer to an Aboriginal and Torres Strait Islander male health and wellbeing program. Descriptive data synthesised, and seven key program elements were analysed: (1) Origin of Design, (2) Governance, (3) Leads/Facilitators, (4) Funding, (5) Length and Frequency, (6) Outcomes and Measures, and (7) Monitoring and Evaluation.

**Results:**

The review identified 54 programs described in 49 publications that were published between 1998 and 2022. Only 20 publications were peer‐reviewed articles. Most programs (*n* = 44) were instigated, co‐designed or adapted by Aboriginal and Torres Strait Islander people to suit cultural and community needs. Reporting on key program elements varied, with only *n* = 25 of the included publications reporting governance systems.

**Conclusions:**

This work is the first to synthesise the literature and describe the key elements of Aboriginal and Torres Strait Islander male health and wellbeing programs. Relatively few publications were found describing programs designed specifically for this population group.

**So What?:**

While the descriptive findings of the programs and their key elements in this review can assist health promotion and primary care practitioners, further investment and research are required to strengthen the evidence base and achieve the best health and wellbeing outcomes for Aboriginal and Torres Strait Islander males.

## 1 INTRODUCTION

More than any other subgroup of Australia's population, Aboriginal and Torres Strait Islander males experience the highest rates of morbidity and mortality across a range of communicable and non‐communicable health conditions.^1^, ^2^ Published in 2023, the Aboriginal and Torres Strait Islander Health Performance Framework report indicated the life expectancy of Aboriginal and Torres Strait Islander males is 4 years less than their female counterparts, and nine and 11 years less than non‐Indigenous males and females, respectively.^3^ Scholars recognise that there should be a greater focus on Aboriginal and Torres Strait Islander male health and wellbeing, including supporting this population to access health programs and services.^4^, ^5^

Despite the elevated need, Aboriginal and Torres Strait Islander males under‐utilise primary health care services (PHCSs),^6^ and several studies have highlighted that males utilise these services less often than their female counterparts.^7^, ^8^, ^9^ Other studies have identified barriers to access including hours of service, appropriate staffing, and the lack of specific or dedicated areas for males within clinics.^8^, ^10^, ^11^, ^12^ When males do present at health services, some have negative experiences, including culturally inappropriate care reducing their likelihood to disclose important health information to clinicians and return to the service,^8^ or engage in the programs they offer. Cultural safety and culturally appropriate staff and services are important factors that enable Aboriginal and Torres Strait Islander males to engage in primary health care programs and services.^8^, ^13^ Aboriginal Community Controlled Health Organisations (ACCHOs) are PHCSs that are led and operated by the local Aboriginal community, and play an important role in providing holistic, comprehensive, and culturally appropriate health care.^14^ In addition to PHCSs, there are a range of other settings where Aboriginal and Torres Strait Islander males engage in health promotion and prevention initiatives. These include health and wellbeing programs delivered on Country or in sport clubs, and these settings are potentially more culturally responsive and meaningful, enticing men to engage.^15^ Men can also express interest to take part in health and wellbeing programs via local councils and charitable organisations (e.g., Australian Red Cross).

A recently published scoping review of literature available prior to June 2015 identified 71 chronic disease risk reduction programs for Aboriginal and Torres Strait Islander people.^16^ There was significant diversity among these programs, as the delivery tended to be influenced by the local context, target community group, and priority need(s) being addressed. The size and scope of the body of literature regarding Aboriginal and Torres Strait Islander male health and wellbeing programs and the key elements of these programs has not yet been investigated. There is little evidence to inform the development and delivery of health and wellbeing programs for Aboriginal and Torres Strait Islander males.

In August 2022, a preliminary search of MEDLINE, the Cochrane Database of Systematic Reviews and JBI Evidence Synthesis was conducted, and there were no current or systematic reviews or scoping reviews underway related to Aboriginal and Torres Strait Islander male health and wellbeing programs were identified. The objective of this review is to collate information about Aboriginal and Torres Strait Islander male health and wellbeing programs, describe the contexts in which they have been implemented, and to catalogue the key program elements reported. By synthesising the available evidence and describing current strengths and opportunities, this scoping review will inform the future delivery of such programs.

## 2 METHODS

### 2.1 Registration and review question(s)

This scoping review was registered on Open Science Framework in August 2022.^17^ To better enhance the relevance of the review for Aboriginal and Torres Strait Islander populations,^18^ the review questions were developed after initial consultations with Aboriginal and Community Controlled Health Organisations (ACCHOs) and Aboriginal Medical Services (AMS):

1. What Aboriginal and Torres Strait Islander male health and wellbeing programs have been reported in the literature?
  - What is the scope of health and wellbeing programs that have been implemented?
  - In what geographical locations and settings have these programs been implemented?
  - To what extent are the key elements of these programs reported?

### 2.2 Inclusion criteria

#### 2.2.1 Participants

The review considered literature that included Aboriginal and Torres Strait Islander males aged 15 years and older. We have used the term ‘Male’ as it is inclusive of all males (i.e., initiated, uninitiated, heterosexual, homosexual, sister girls, transgender).^19^ Studies with participant populations of boys (i.e., males under 15 years of age) and men were considered, if the majority of participants were aged over 15. Participant populations that included non‐Indigenous people were considered eligible for inclusion only if Aboriginal and Torres Strait Islander males were the primary focus. Programs designed for Aboriginal and Torres Strait Islander families or communities were excluded. However, programs explicitly aimed at supporting fathers were included.

#### 2.2.2 Concept

The review considered literature that included a health program designed for, and delivered to, Aboriginal and Torres Strait Islander males. The health program must aim to improve Aboriginal and Torres Strait Islander male health and/or social and emotional wellbeing—a holistic concept of health encompassing several Aboriginal and Torres Strait Islander domains and determinants, beyond physical health indicators.^20^

#### 2.2.3 Context

The review considered literature with Aboriginal and Torres Strait Islander males that were set in any Australian context and setting, such as local communities, PHCSs, ACCHOs, hospitals, correctional facilities and rehabilitation centres. No specific contexts or settings were excluded.

#### 2.2.4 Types of publications

This scoping review considered all peer‐reviewed qualitative, quantitative, economic and mixed methods studies. Observational, experimental, quasi‐experimental study designs and descriptive cross‐sectional studies were considered. Editorials, perspective, expert opinion, policy, and discussion papers were not considered. Systematic reviews or literature reviews were also excluded.

Grey literature considered were web pages, program evaluation and cost analysis reports. Other types of publications, including videos, newspaper articles and advertisements were not considered.

### 2.3 Search strategy

The search strategy is shown in Appendix A. It was developed with the assistance of a research engagement librarian with the aim to locate both published and unpublished studies.

A comprehensive search was then conducted across CINAHL, Informit, OvidMedline, Proquest, Psychinfo, Scopus, and WebOfScience. Key search terms included ‘Aboriginal’, ‘Torres Strait Islander’, ‘Indigenous’, ‘First Nation’ ‘men’, ‘male’, ‘health’, ‘wellbeing’ and ‘program’. The search strategy, including all identified key terms, were adapted for each included database. Reference lists of included studies were checked for additional studies, as well as utilising expert researchers and librarians for any further material. Searches were conducted across identified grey literature databases to locate published work or projects in progress, including Australian Indigenous Health*InfoNet*, the Lowitja Institute, Healthy Male, Australian Men's Sheds Association, Australian Men's Health Forum, Beyond Blue and Movember. Websites were identified through consultation with expert Aboriginal and Torres Strait Islander advisors.

See: Appendix A. Search strategy.

### 2.4 Study selection

All identified articles were downloaded onto Zotero^21^ before being uploaded into Covidence systematic review software,^22^ where all duplicates were removed. KC screened title and abstract of 20 articles to pilot test the search and demonstrate the screening process to co‐authors. Next, KC, RN and LN independently screened title and abstracts of articles against the inclusion criteria to identify articles for full‐text screening. Publications submitted for full‐text screening were retrieved via the Flinders University library and Google Scholar. When full texts were not available, access requests were made to the Flinders University library. Simultaneously, emails were sent out to corresponding authors or organisations (grey literature) requesting access to the articles. Authors CG and RN independently screened full text articles against the inclusion criteria, with any conflicts resolved by consensus following discussion with KJC and/or KC. Reasons for exclusion of publications at full text that did not meet the inclusion criteria were recorded. The search and inclusion process was reported through a Preferred Reporting Items for Systematic Reviews and Meta‐Analyses extension for scoping reviews (PRISMA‐Scr) flow diagram.^23^

### 2.5 Data extraction

Following consensus of the included publications, CG and RN extracted the data independently using a modified Covidence template. Data extracted included the program name, scope, and aim, the funding source, who initiated, led and facilitated the program, governance structures, setting, geographical location, participants, program activities, length and frequency, key findings, outcomes and measures and whether the program had been evaluated. Extracted data was then cross‐checked in discussion by RN and CG to reduce the likelihood that relevant data was missed or incorrectly extracted.

### 2.6 Data analysis and presentation

The included articles and details of each identified Aboriginal and Torres Strait Islander male health program were summarised narratively in a table. The geographical location in which programs were delivered were determined using the Modified Monash Model (MMM) of classification codes for levels of remoteness.^24^

#### 2.6.1 Aboriginal and Torres Strait Islander male health key program elements

**FIGURE 1:**
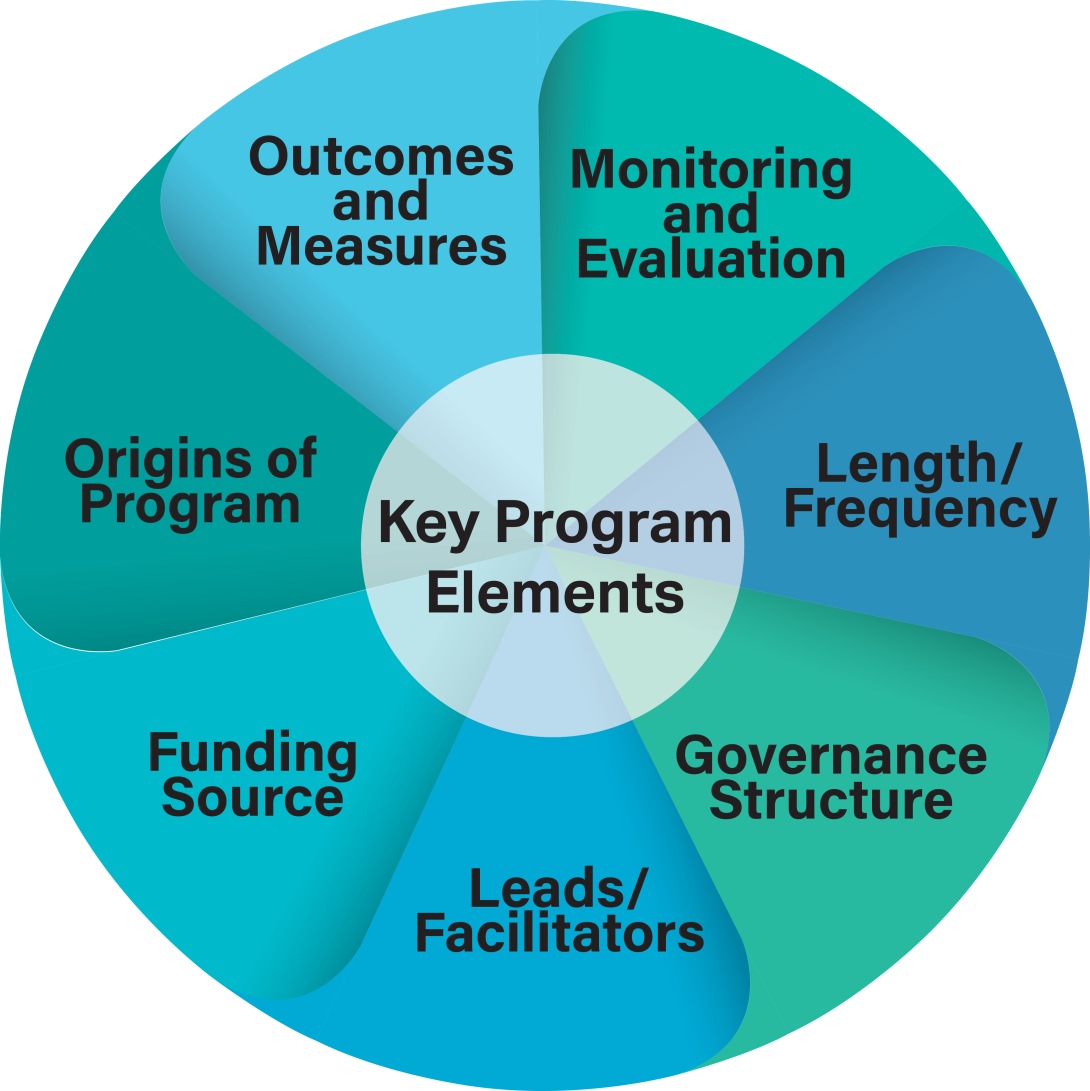
Key program elements.

In the absence of an evidence‐based framework for the design and reporting of Aboriginal and Torres Strait Islander male health and wellbeing programs, the research team developed a model of key program elements that were used as a deductive analysis tool in this scoping review (Figure 1). The seven program elements are (1) Origin of Design, (2) Governance, (3) Leads/Facilitators, (4) Funding, (5) Length and Frequency, (6) Outcomes and Measures and (7) Monitoring and Evaluation. This framework was informed by seminal work,^25^, ^26^, ^27^, ^28^, ^29^, ^30^ discussions among thought leaders (KC, KJC and GG), and established criteria and guidelines by research funders, such as the Australian Research Council, the Medical Research Future Fund and the National Health and Medical Research Council.^31^, ^32^ The model provided a structured approach for reviewing and charting pertinent information in addressing our research question (1c), illustrating seven key inter‐related elements of Aboriginal and Torres Strait Islander male health and wellbeing programs. We described how these key elements were reported within the included publications in Table 2.

## 3 RESULTS

### 3.1 Characteristics of included publications

**FIGURE 2:**
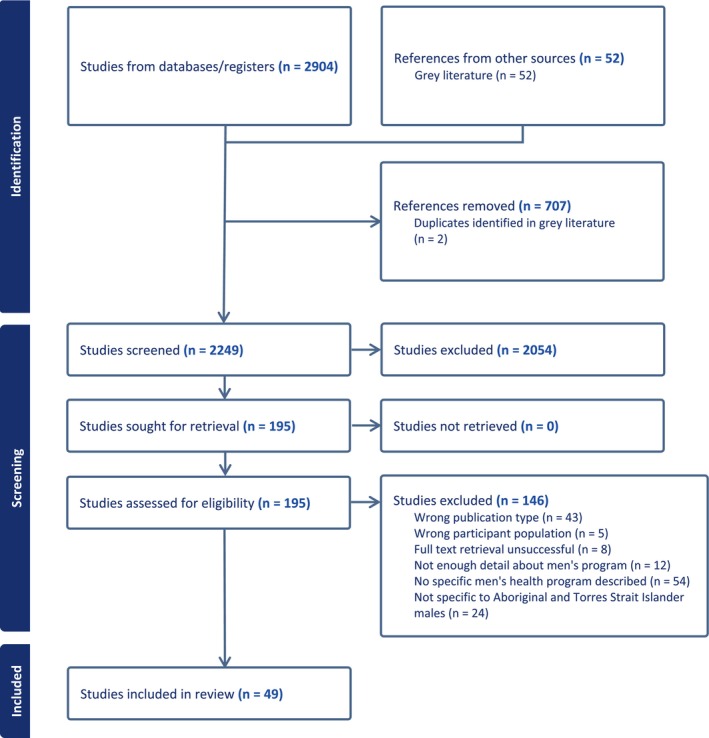
PRISMA flow chart detailing results of the literature search and study inclusion.

The PRISMA flow diagram is shown in Figure 2. The literature search was completed between October 2022 and March 2023. After removing duplicates, a total of 2249 publications were screened for inclusion of which 49 met the inclusion criteria (2.2%). The main reason for excluding literature was the absence of a health program designed for, and delivered to, Aboriginal and Torres Strait Islander males (*n* = 54). Included publications were from peer‐reviewed journal articles (*n* = 20), grey literature reports, conference abstracts, a newsletter, fact sheet and resource guide (*n* = 23), web pages (*n* = 4) and book/chapters (*n* = 2). Publication dates ranged from 1998 to 2022, most articles were published in or after 2000 (*n* = 45), and only nine articles were published in the last 6 years.

### 3.2 Characteristics of Aboriginal and Torres Strait Islander male health programs

This scoping review identified 54 Aboriginal and Torres Strait Islander male health and wellbeing programs from the included 49 publications. A narrative description of each program and their characteristics are shown in Table 1.

**TABLE 1:** Characteristics of included publications and programs.

Program name, citations and type of publication	Program scope	Geo‐location and setting	Participants	Activities/summary/style of delivery	Key findings
Koori^a^ Men Exploring New Directions (MEND) Group. *Anderson 1998 (Conference paper)*. *Anderson and Telford 2000 (Report)*.	Anti‐violence program, including behaviour change and fitness. Assists men to find their place in society and be proud of their culture and selves.	Lismore, NSW—MM 3. Gippsland, VIC—MM 5. Men's group. Setting NR.	Aboriginal (Koori) men. Referred or voluntary. Average of 7 men per group/ week.	Relaxed, unstructured, informal, supportive group sessions, beginning with a 4‐point ‘check‐in’. Uses educational methods. Hand‐outs on identifying different violent and non‐violent behaviours. Storytelling, listening and exploring anger using skills and attitude exercises (e.g., Power and Control Wheel). Father and son camps which include Elders.	The anti‐violence MEND Aboriginal group has similar rates of success to the non‐Aboriginal MEND groups.
Well men's check‐ups. *Smith and King 1998 (Peer‐reviewed journal article)*.	Health promotion and screening program.	East Arnhem Land, NT—MM 6. Sporting occasions such as football training or games.	Local Aboriginal men attending sporting occasions, football training/games.	The health centre taken to the people (sporting occasions). Health education, lifestyle role models, Men's health check‐ups/screening. BBQ after check‐ups. Questionnaire identifying lifestyle habits and stress/distress.	Concept of bringing the health clinic to the people was successful. Concentrated resources at football settings. Use of positive role models, football player that spoke the language of that community. Stated that these checkups can be modified for other population groups.
GutBusters. *Egger* et al *1999 (Peer‐reviewed journal article)*.	Men's weight loss program.	8 island communities in the Torres Strait region, QLD—MM 7. Various locations; beaches, community halls, canteens, or sitting outside.	135 Torres Strait Islander male participants aged 22–60 years (mean age 40 years).	Education on behaviour and lifestyle, changing ‘obesogenic’ habits (eating well, more fibre, less fat, more exercise). Men's waist circumference measured at 2, 6 and 12 months.	Of the 45 men followed‐up over 12 months, 4 gained weight and nine remained stable. Average weight loss over 1‐year period was 3.3 kg or 3.5% (range 716.0–5.5 kg). Average waist loss was 4 cm or 3.5% (range −14 to +5 cm).
Indigenous Young Fathers' Support Group. *Jia 2000 (Peer‐reviewed journal article)*.	Support for young Indigenous fathers.	Brisbane, QLD–MM 1. Group sessions at strategic locations requested by the young fathers.	Young Indigenous fathers aged between 16 and 25 years.	The program was participant‐led, as such the young fathers were encouraged to design the content, process, structure and place of the group. Education about physical, emotional, spiritual and cultural needs of infants and/or children; their parenting role and maintaining cultural values. The group members established rules for the group: alcohol and drug free, respect each other's opinions and no criticisms, no obscene language, our families are welcome, no arguing or fighting, confidentiality, and multi‐cultural.	Group facilitators reported program acceptance and engagement by the young fathers. Meeting notes showed the participants discussed ownership of the project, desired content, processes, structure, and place of the men's group. The young fathers started an indoor cricket team after participating in the program.
Sex offender program. *McCallum 2000 (Conference paper)*.	Sex offender program (intervention).	Alice Springs, NT—MM 6. Prison program.	Traditional Indigenous men, mainly from remote communities, who had committed sex offences.	Group sessions using culturally appropriate intervention methods.	The program was well received by traditional Indigenous offenders within NT prisons. Early indications showed it may be useful in preventing re‐offending.
Yarrabah Men's Health Group. *Tsey* et al *2002 (Peer‐reviewed journal article)*. *Tsey 2019 (Book)*.	A holistic healing Program.	Yarrabah, QLD—MM 5. Men's group. Setting NR.	Aboriginal men living in Yarrabah area at risk of suicide including men who are heavy drinkers, have a history of violence and men in the corrections systems. 7–12 Aboriginal men (regulars) over 2‐year period of operation.	Men's group to restore their rightful role in the community using a holistic healing approach encompassing in the program the spiritual, mental, physical, emotional and social aspects of life. Activities: Health education sessions, men's health clinics provided by a visiting Aboriginal doctor, activities to promote social skills and bonding (meals in restaurants, visiting the cinema, hunting, fishing and camping).	The group identified four initiatives to explore: 1. Personal Development, Leadership and Parenting, 2. Employment, 3. Education and Training, 4. Tradition, Culture and a Yubba Bimbie Men's Place. In a 9‐year period, since 1998, when the Men's Group formed, suicide rates in the community dropped from 3 to 4 suicides per year to 2.
The Footy Fitness Project. *Davis 2001 (Report)*.	An innovative health promotion strategy linked to sport for health screening and education. Introducing young men to clinic staff, raise health awareness and improve access to services.	Tennant Creek, NT—MM 7. Team training sessions and health clinic.	Aimed at young Aboriginal men. 92 men accessed the program. Age range: 14–35 years and over.	Screening assessments at Clinic, or Football match days. Adult health checks, screening for risk factors, check/update immunisations, counselling/advice on alcohol, tobacco and other drug use. Addressed fitness and injury prevention with players and coaches.	A high prevalence of cardiac risk factors was identified in this study. This finding is consistent with the known high prevalence of ischaemic heart disease in Indigenous Australians suggesting that modifiable risk factors should be targeted at an early age.
Wadja Warriors—healthy weight program. *Smith 2002 (Peer‐reviewed journal article)*.	Healthy weight promotion program. Increasing knowledge of, and promoting nutrition, healthy lifestyles and physical activity.	Woorabinda, QLD—MM 6. Setting NR.	Football team/club members as well as men in the wider community.	Workshops teaching skills required for healthy lifestyle changes (cooking, food safety/hygiene, shopping better, reducing fat intake and sports nutrition). Screenings for diabetes and other conditions. Questionnaire at the start and end of the program.	Participants reported making changes to their eating habits, way of cooking and increased physical activity they did. This was reflected in the reported changes to specific food habits and other behaviours examined by the questionnaire.
Empowering men to be who we know they are. *Krieg 2004 (Newsletter)*.	Empowerment program addressing domestic violence.	Point Peron, WA—MM 1. Three‐day camp at Port Peron beach.	18 Aboriginal men aged 18–72 years.	Loosely structured group talks about what things have impacted their lives. Team building skills and social activities.	Program made a difference to attendees' attitudes around their behavioural problems (although) numbers not reported
The Countrymen Program. *Partnerships Against Domestic Violence (Australia) 2004 (Report)*.	Pre‐ and post‐release prison program.	Cairns, QLD—Regional Centre MM 3. Lotus Glen Correctional Centre and in Community	Indigenous male inmates (pre and post release) and Indigenous men in community.	None specified. Began as a pre‐ and post‐release prison program and was later redesigned to provide training for Indigenous support workers and counsellors to work at a community level.	NR.
Counselling program for Indigenous men who use violence. *Partnerships Against Domestic Violence (Australia) 2004 (Report)*.	Counselling program for Indigenous men who use violence. Addressed issues of responsibility, empathy and alternative behaviour.	Geo‐location NR. Family and Children's Services, WA.	Indigenous men who use violence.	Culturally appropriate counselling programs over 12–14 weeks; and, where appropriate, family involvement.	NR.
Ending Family Violence Program. *Partnerships Against Domestic Violence (Australia) 2004 (Report)*.	Ending family violence program.	Geo‐location NR. Aboriginal and Torres Strait Islander Unit, Department of Corrective Services, QLD.	Indigenous men who had been convicted of a violent offence.	A therapeutic and educative focus. Offenders were assisted to raise self‐esteem; build confidence; identify problems; and recognise/learn strategies and alternatives to violence.	NR.
HomeGround's Koori Men's Outreach Recreation Program. *Hoare 2006 (Peer‐reviewed journal article)*.	Outreach recreation Program. Aimed to create a sense of special time and place for the men.	Collingswood Melbourne, VIC—MM 1. Setting NR.	Koori men from the Collingwood Koori community.	Recreational activities in nature. Day trips: Fishing, fires, cooking.	Men ‘re‐create’ themselves engaging with aspects of themselves that are not stimulated by the often‐brutal inner urban environment (drugs, alcohol and homelessness).
Yaba Bimbie Indigenous Men's Support Group. *McCalman* et al *2005 (Report)*. *McCalman* et al *2007 (Peer‐reviewed journal article.)*	Family wellbeing, violence and suicide prevention program.	Yarrabah, QLD—MM 5. Men's Group at Gurriny Yealamucka (Aboriginal health service).	Aboriginal and Torres Strait Islander men in Yarrabah.	Discussion and support group, with sessions based on one of the topics of the Family Wellbeing Program. Occasional guest speakers. Meetings provide informal support to men for relationship issues, parenting, sorry business and community issues. A holistic healing approach, encompassing the spiritual, mental, physical. emotional and social aspects of life. Participants received awards to recognise their achievements and abilities.	Men's group role in helping prevent suicides and combat family violence, identifying the root problem, reclaiming traditional culture integrated with Christian spirituality. Suicide rates dropped at Community level.
Ma'Ddaimba‐Balas Indigenous Men's Group. *McCalman* et al *2006 (Report)*.	Men's support group. Health and wellbeing.	Innisfail, QLD—MM 4. Men's Group held at the Mamu Aboriginal Medical Service.	Most meetings attract 10–15 participants, aged between 25 and 40 years. Local Aboriginal and Torres Strait Islander men, including men referred by the court.	Support for Indigenous men in the courts. Multiple level interventions; education programs and counselling (substance abuse, anger management, ending offending and domestic violence, family wellbeing), improving health in partnership with Mamu Medical Service, working with Indigenous youth, sport and social events.	The most commonly mentioned community impact was the effect that Men's Group social and sporting events have had on increasing community participation and cohesiveness, both within Indigenous families and with the wider community.
Koori Fathering Program. *Newell* et al *2006 (Report)*.	Pilot program aimed at supporting fathers.	Northern Rivers Area, NSW—MM 1, 3–4. Men's group (at the Northern Rivers Area Health Service).	25 local men (fathers), aged between 18 and 53 years.	15‐week course to support Aboriginal men and, indirectly, their partners and children. Topics include positive relationships with children/partners; children's development; responsibility of fatherhood; communication skills.	Participating men indicated (self‐rated) overall improvements in parenting skills, seeking support, relationships with partners/children and in the wider community.
Rekindling the Spirit Program. *Australia. Human Rights and Equal Opportunity Commission 2008 (Report)*. *The Healing Foundation and White Ribbon 2017 (Report)*.	Empowerment and holistic healing program focussing on Indigenous health and wellbeing.	Lismore, NSW—MM 3. Men's group (Urban and Rural locations). Setting NR.	Many participants are referred by the Department of Corrective Services or Department of Community Services.	Provides support in casework, advocacy and referrals. Major themes: violence; alcohol and drug use; emotional connection with self; trauma and abuse; relationship with partner and children; practical skills; financial supports; connecting with Aboriginal culture. Supported pathway to mainstream service providers, including primary and mental health services as well as training and employment providers. Men's camps and father and son camps (fishing, hunting, cultural activities).	NR.
Yerli Birko (Men's Group). *Australia. Human Rights and Equal Opportunity Commission 2008 (Report)*.	Men's Group. Family violence prevention program.	Adelaide, SA—MM 1. Men's shed group. Setting NR.	A rotating core group of 10–15 men. 170 men took part in Yerli Birko activities within the first 3‐months with 590 men attending one or more of the group's activities in the first 12‐months	Family violence (and other issues) are addressed though Talking Circles, usually led by an Elder or facilitator. Cultural and social activities, healthy food education, sharing stories, preserving and reviving male culture.	NR.
Spirited Men. *Australia. Human Rights and Equal Opportunity Commission 2008 (Report)*.	Men's support group.	Kalparrin Community, Murray Bridge SA—MM 3. Men's group. Setting NR.	Between 10 and 25 men from across the region. Men entering the Kalparrin rehabilitation program must attend this men's group.	Information sharing about support services available in the area. Discussion about the role of men in the local Indigenous community and strong anti‐family violence messages. Counselling is also offered.	NR.
Tau Nagaraldi—Anger management and family violence program. *Australia. Human Rights and Equal Opportunity Commission 2008 (Report)*.	Anger management and family violence program.	Kalparrin Community, Murray Bridge SA—MM 3. Men's group. Setting NR.	40–45 men have had regular contact with the group in an area with only 241 Indigenous males over 15 years of age.	Utilises narrative therapy techniques to help the men understand their anger. It does not invalidate their feelings (for example, of injustice at racism) but aims to teach the men to stop and think before acting on their anger.	NR.
The Mount Isa Murri^b^ Court—Men's support group *Australia. Human Rights and Equal Opportunity Commission 2008 (Report)*.	Murri men's support group to assist with family violence offences, providing alternatives to detention through culturally appropriate intervention.	Mt Isa, QLD—MM 7. Men's group. Setting NR.	Mandated men who have pleaded guilty to Family violence offence.	There is no strict program outline, but an opportunity for men to ‘come together and ‘yarn’ about some deep issues in safe place’ (ways to manage anger and general anti‐violence messages). Older Indigenous men attend to mentor and offer support to participants from court. Occasional attendance by Aboriginal health workers to provide health checks and health education, and local alcohol and drug services to promote their program and provide information about harm minimisation.	Facilitator of the group observed family violence dropped significantly among men who have worked with his group and the Murri Court and estimated that 90% of the men have not reoffended again.
The Koori Cognitive Skills Program (KCSP). *Jones and Atkinson, 2008 (Book chapter, 13)*.	KCSP offered in prison and community correctional settings. Adapted for Aboriginal and Torres Strait Islander prisoners and offenders.	Barwon Prison, a maximum‐secure men's prison located near Geelong, VIC—MM 1. Loddon Prison, medium‐secure men's prison in regional VIC—MM 4.	No screening criteria beyond Indigenous status and willingness to participate. The groups ranged in size from 5 to 10, and a total of 24 participants completed the program.	Role plays that reflect family and community pressures; Talking circles/Discussion topics (stolen generations, deaths in custody, health issues and family concerns/violence); Visual and sensory modes of learning; Cultural activities (e.g., shield carving, language learning).	NR.
The Miller Aboriginal Men's Group Bike Fleet. *Bindon* et al, *2009 (Peer‐reviewed journal article)*.	A cycling promotion program to help address health and social issues in the community.	Liverpool/Fairfield area, NSW—MM 1. Setting NR.	7 men from the Miller Community Centre, Aboriginal men's group.	6 bikes were provided and could be used for purpose‐related travel, physical activity, recreation. Group rides were conducted to enhance cycling proficiency and confidence. The Aboriginal Men's Group was invited to attend an accredited Bike Fleet Maintenance course. 3 bike maintenance workshops were also held.	The project was popular with members of the Miller Aboriginal Men's Group. 7 Aboriginal men completed the bike fleet maintenance course and 5 participated in the cycling proficiency group rides.
‘Mibbinbah’ Indigenous Men's Shed. *Bulman 2009 (Peer‐reviewed journal article)*.	Mibbinbah's Indigenous Men's Sheds Program addresses depression and anxiety and looks to improve the health of Aboriginal and Torres Strait Islander men by creating safe spaces.	Location not specified. A total of 7 sites in the NT, VIC, QLD and NSW. Men's shed group. Setting NR.	NR.	Mibbinbah, ‘eagle men place’ or ‘men's place’ is an innovative program committed to improving the health of Aboriginal and Torres Strait Islander males by providing safe spaces, education and support to empower individuals.	Members have increasingly overcome stigma by talking more openly after having breathed a collective sigh of relief to hear from visiting specialists that depression and anxiety are not psychiatric illnesses.
Brothers inside. *Hammond 2011 (Peer‐reviewed journal article)*.	Fathering program.	Location undisclosed. Prison setting.	Aimed at Aboriginal prisoners. Ages range from 18 to mid 50 years.	Discussion groups and workshops including creative activities (drawing and writing poetry). Topics: importance and strengths of fathers, believing in themselves, resilience and ways of strengthening connections with their children.	Older and experienced fathers offered valuable insights to the group. Frequent disruption to workshops as a result of prison setting (e.g., lockdowns), and varying levels of attendance due to men having appointments, being released or transferred.
The Shed in Mount Druitt. *Macdonald and Welsh, 2012 (Report)*.	Empowerment and support group.	Mount Druitt, NSW—MM 1. Men's shed group. Setting NR.	Aboriginal and Torres Strait Islander males in the Mount Druitt area.	The Shed is a one‐stop shop for men and their families to obtain information and help on access to services (legal aid, health services, mental health, social services) and enjoy ‘a feed and a yarn’.	Suicide rates in the area have decreased since the opening of the Shed, and there has been an increase in attendance of men at The Shed.
Alive and Kicking Goals! *Tighe and McKay 2012 (Peer‐reviewed journal article)*.	Social and emotional wellbeing (SEWB), suicide prevention and peer education program.	Broome, WA—MM 6. Broome Saints Football Club (BSFC).	Young Indigenous men at the BSFC club. Also included non‐Indigenous men however they were a minority. 644 participants attended at least one activity. Of these participants, 421 were Indigenous. 16 men were trained as Peer educators in suicide prevention.	Weekly training sessions in holistic suicide prevention for Peer Educators. Other activities included training programs such as ‘ASIST’ and ‘Who You—Which Way’.	Peer educators learned practical skills in suicide awareness and prevention. Findings showed various co‐design strategies for establishing Aboriginal and Torres Strait Islander health programs, including utilising sports settings to strengthen identity, community connectives and empowerment; and building upon pre‐existing community groups/structures.
Jumna Wal—Creating Better Pathways for Men. *Western Sydney University 2012 (Fact sheet)*.	Empowering Aboriginal and Torres Strait Islander men against recidivism.	Hawkesbury, Western Sydney, NSW—MM 1. Men's Health Information and Resource Centre based at the University of Western Sydney.	Aboriginal and Torres Strait Islander men.	Teaches life skills and better pathways. Links men with important service providers in the areas of housing, employment, health services, the legal system and other bodies. The program concludes with a three‐day cultural camp.	NR.
Tweed Yarn Up Group. *Newell 2013 (Report)*.	Overall SEWB program for Indigenous men.	Tweed Heads, NSW—MM 1. Men's group. Setting NR.	33 Indigenous men, aged 17–60+ years.	Yarn Up Group covers topics, such as: Violent behaviour and impacts, Fathering and child development, Anger management, Self‐awareness, Goal setting, Relationship and listening skills.	An increased level of self‐confidence, feeling less isolated, increased awareness of own behaviour, and so on.
The Koorie Men's Health Day. *Isaacs and Lampitt 2014 (Peer‐reviewed journal article)*.	Health promotion. Encouraging Koorie men (Aboriginal men from Southeast Australia) to seek help for their mental illness and offers support to continue treatment.	Gippsland, VIC—MM 5. Setting NR.	Aimed at Koori men over the age of 18 years. 17 Koorie men aged between 16 and 65 years.	BBQ, health check and screening for mental health problems. Information sessions by male academics on cardiovascular disease, diabetes and depression. Psychological distress measured using the Kessler 10 questionnaire (K‐10).	Of these 17 participants who were screened using the K‐10 questionnaire, seven had a significant score of 25 or higher.
Gatharr Weyebe Banabe Program. *Mosby 2014 (Peer‐reviewed journal article)*.	Healing program addressing violent behaviour.	Rockhampton, QLD—MM 2. Setting NR.	Referred men who have perpetrated family violence.	The program consists of phases: Preparation for ceremony, Contemporary healing ceremonies, Ongoing healing pathways. Case management, counselling and group work.	NR.
The sport and active recreation programs in an Indigenous men's shed. *Cavanagh* et al. 2015 *(Peer‐reviewed journal article)*.	Sport and recreation program to increase health, attitudes, social connectedness within their community, and sense of belonging.	Geo‐location and setting NR. Men's shed group (remote location).	Indigenous men from a remote community men's shed aged between 22 and 65 years. 15 men attended the swimming and water aerobics. 15 men attended gym activities. 30 men joined the darts and pool comp. Twenty men (and women) attended the healthy eating program.	The program included swimming, water aerobics, gym‐based activities, darts and pool competitions, combined with a 10‐week healthy eating program.	The Shed became a focal point for the community, re‐connecting cultural harmony, less re‐offending, domestic violence, and addictions. Participants reported eating better, drinking less alcohol, enhanced self‐esteem and health benefits.
Maambart Maam For Maali Moort Wellbeing Pilot Program. *Kickett‐Tucker* et al. *2015 (Report)*.	Wellbeing program for fathers (during the perinatal period). The program took a strength‐based, empowering approach.	Perth, WA—MM 1. Various settings (see activities).	Aimed at new Aboriginal fathers and male carers. 51 participants, all up (different number per activity).	Addressed issues associated with trauma, grief and toxic stress. Key activities include: Open Family Day, Men's BBQ, Men's Yarning Circle, Family Camp, Kayaking, Art Therapy, Super Golf, Men's Camp.	Of the 32 participants who completed both pre‐ and post‐program questionnaires, 9 reported no change in their mental health, 9 reported an improvement in their mental health at post‐program, and 14 reported a decline in their mental health post‐program.
A 12‐week sports‐based exercise programme. *Mendham* et al *2015 (Peer‐reviewed journal article)*.	Exercise program.	NSW regional community. Local fitness centre.	26 participants. Men of Australian Indigenous ancestry, not diagnosed with pre‐existing cardiovascular disease or metabolic disorders.	Supervised sports and gym exercises in a group environment. This study assessed the effect of a 12‐week sports‐based exercise intervention on glucose regulation, anthropometry and inflammatory markers associated with the prevalence of type 2 diabetes mellitus in Indigenous Australian men.	The exercise condition decreased insulin area under the curve (25 ± 22%), increased estimated insulin (35 ± 62%) and decreased insulin resistance (9 ± 35%; *p* < 0.05), compared with control (*p* > 0.05).
Our Men, Our Healing. Wurrumiyanga—Tiwi Men's Healing Program. *The Healing Foundation 2015 (Report)*. *Our Men Our Healing. The Healing Foundation and White Ribbon 2017 (Report)*.	Men's healing program. Cultural and spiritual healing needs for men to deal with Family Domestic Violence (FDV) issues and problems such as AOD.	Tiwi, NT—MM 7. Men's shed group in Wurrumiyanga Community.	An average of over 100 Indigenous men aged between 16 and 60+ years of age. Some men are referred in relation to FDV Orders.	Men engaged with primary and mental health services through the program. Counselling, family support, advocacy and cultural brokerage and case management. Day trips and activities (fishing, spear making, hunting, art), camps, cultural education, yarning groups and community events.	Reported decrease in incidence of family and domestic violence and less violence generally in communities. Reduced observable rates of self‐harm and suicide during the life of the program in two of the three communities.
Our Men, Our Healing. Gurrutu Raypirri Men's Healing Project. *The Healing Foundation 2015 (Report)*.	Men's healing program.	Maningrida, NT—MM 7. Setting NR.	30 men aged between 16 and 60+ years of age. Aimed at men with high levels of FDV and interaction with the justice system, high AOD abuse and poor health and wellbeing.	Counselling, family support, advocacy and cultural brokerage and case management. Day trips and activities (fishing, spear making, hunting, art), camps, cultural education, yarning groups and community events.	Reported decrease in incidence of family and domestic violence and less violence generally in communities. Reduced observable rates of self‐harm and suicide during the life of the program in two of the three communities.
Our Men, Our Healing. Ngukurr Men's Cultural Healing Program. *The Healing Foundation 2015 (Report)*.	Men's healing program.	Ngukurr, NT—MM 7. Setting NR.	98 older men over 18 years. 106 younger men under 18 years.	Counselling, family support, advocacy and cultural brokerage and case management. Day trips and activities (fishing, spear making, hunting, art), camps, cultural education, yarning groups and community events.	Reported decrease in incidence of family and domestic violence and less violence generally in communities. Reduced observable rates of self‐harm and suicide during the life of the program in 2 of the three communities.
Quop Maaman: Aboriginal Fathering Project. *Collard* et al *2016 (Report)*. *The Healing Foundation and White Ribbon 2017 (Report)*.	Fathering program.	Perth, WA—MM 1. Setting NR.	6 Aboriginal men.	A series of workshops themed around Aboriginal fathering, within a Noongar cultural framework. A safe space for older men to share knowledge with younger men. Aimed to raise self‐esteem and build practical skills. Participants also co‐designed a set of workshop resources for Aboriginal fathers. 6 video resources and 7 information sheets.	NR.
The Blue Mountains Aboriginal Men and Youth Program. *Hunter 2016 (Report)*.	SEWB program for Aboriginal and Torres Strait Islander men and youth.	Blue Mountains, NSW—MM 2. Various locations. Settings NR.	Aboriginal and Torres Strait Islanders men, aged between 18 and 24 years of age. Older Aboriginal men also participated in activities as mentors and Elders.	Aboriginal Men's forums, cultural camps, art therapy, wellbeing and mental health day. Workshops and activities providing educational support with literacy and numeracy, further education, skills development, employment, housing, health and referrals to facilitate access to social services. Regular Elders golf activities.	NR.
Right Tracks Program. *Central Australian Football Club 2017 (Website)*.	Sport and mentoring program.	Alice Springs, NT—MM 6. Football Club.	Aimed at 18–25‐year‐old Aboriginal men disengaged from society. Must be in full time employment, study or in the Right Tracks Program to be eligible to play in the Central Australian Football Club (Redtails).	Numeracy and literacy, health and wellbeing, mental health, alcohol and tobacco, education, language and culture, job ready mentoring, challenge projects, work experience/pathways, life skills training, pathways to higher learning, apprenticeships and leadership development.	NR.
Stayin’ on Track. *Fletcher* et al *2017 (Peer‐reviewed journal article)*.	Young fathers' support via online and mobile phone‐based resources.	Undisclosed: one regional city and two rural towns (1 small and 1 large) in NSW. Setting not applicable.	20 young Aboriginal fathers aged 18–25 years old.	Social, cultural and emotional support to the fathers to address issues in relation to mental health and wellbeing—via Internet‐ and mobile phone‐based resources.	Increased information seeking, support, mental health, knowledge around fathering and decreasing stress.
*Illawarra Koori Men's Support Group*. *Interdisciplinary Indigenous Health Research Group and Illawarra Koori Men's Support Group. 2017 (Report)*.	Men's Support Group.	Albion Park, NSW—MM 1. Men's group. Setting NR.	5333 men attended the group between 2015 and 2017.	Culturally appropriate education programs and services focussing on physical and SEWB. Arts, crafts and youth mentoring. The men's group is also involved in a range of local community activities such as working with local schools and holding community BBQs.	NR.
Strong Men Strong Communities—Men's healing project. *The Healing Foundation 2016 (Report)*.	Men's healing program with a focus on developing and strengthening men's SEWB, leadership in their family and community.	Darwin, NT—MM 2. 7 Darwin town communities. Country camps, meetings, BBQ (East Point, NT) and workshops (1 on Crab Craw Island, NT).	68 Aboriginal and Torres Strait Islander men aged 17–60+ years of age. 60 per cent of attendees can be described as ‘regular’, attending the majority of activities and workshops.	Activities and workshops included cultural healing, music, drug education, relationships and communication education, parenting, health and hygiene, pre‐employment program, mentoring programs. Back to country camp.	More men are seeking SEWB support and feeling supported; are empowered and job ready or working; have stronger identity, reconnecting to and respecting culture. There is more positive communication within community and participation by men.
The Indigenous Men's Service. *The Healing Foundation and White Ribbon 2017 (Report)*.	Family violence prevention program.	Darwin, NT—MM 2. Setting NR.	Men from Darwin town camps.	Healing activities. Education on drug and alcohol, relationships and communication, parenting, health and hygiene and a pre‐employment program.	NR.
Dardi Munwurro's men's healing programs: Strong Spirit Program. *The Healing Foundation and White Ribbon 2017 (Report)*.	Behaviour change program.	Geo‐location NR. Victorian correctional facilities.	NR.	Strong Spirit program supports men in healing trauma using cognitive therapy with cultural healing.	NR.
Dardi Munwurro's men's healing programs: The Men's Healing and Behaviour‐Change program. *Deloitte 2021 (Cost analysis report)*.	Family violence prevention service, healing and behaviour change program, addressing the drivers for violence by strengthening culture, developing pride and encouraging healthy relationships.	Undisclosed. Locations in Melbourne (MM 1) and across VIC. Activities occur on traditional lands or at Aboriginal and Torres Strait Islander community‐controlled premises.	Open to all Aboriginal and Torres Strait Islander men. It is also delivered as a 1‐week intensive program for incarcerated Aboriginal and Torres Strait Islander men who have been referred.	Men's group counselling sessions centred around themes such as intimacy, responsibility, communication, emotions, leadership, and relationships.	80% reduction in self‐reported episodes of violence. Significant drop in alcohol and other drugs use post program. Rise in employment outcomes post program. Very significant drop in incarceration, court orders and community corrections orders post program.
Dardi Munwurro's men's healing programs: Ngarra Jarranounith Place (NJP). *Deloitte 2021 (Cost analysis report)*.	Residential family violence prevention and healing program.	Melbourne, VIC—MM 1. Setting NR.	The program accepts men on Family Violence Intervention Orders, those charged with family violence offences in the previous 12 months, court‐ordered referrals, and self‐Referrals from Dardi Munwurro's prison program.	NJP offers accommodation and structured weekly programs that include a vocational program with Parks Victoria, music, writing, art, and other cultural programs, including smoking ceremonies, yarning circles, and meditation. One‐on one support and group activities. Medically supervised detoxification (if required).	Stronger cultural identity, cultural knowledge, and community connections, and positive identity. Noticeable improvements in clients' ability to manage their emotions, understand the impact that violence has, and accept responsibility for violent action.
Breakthrough Violence Program—Changing Minds and Saving Lives. *Lechleitner* et al. *2018 (Report)*.	Addresses men's violence.	Alice Springs, NT—MM 6. Men's Shed. Setting NR.	25 men (for each pilot trial ×2) aged between 20 and 40 years. Men participating in Aboriginal Alcohol Program Unit as early prisoner release, self‐referrals, or referred by the courts.	Uses ‘Mentalisation’, a psychoanalytic approach. Program topics include the nature, pattern and risk factors of violence. Emphasis of mind, developing empathy and tools for control.	NR.
Code 4 Life: Arraty Angka—Straight Talk. *Desert Knowledge 2019 (Website)*.	Reformation and personal change by delivering workshops reconnecting Aboriginal men with their cultural responsibilities and find strength in one's role in their community.	6 Central Australian locations, NT—MM 6 and 7. Workshops.	136 Aboriginal men from 6 central Australian regions, aged 18–50 years of age.	Workshops conducted in 6 communities. Various engagement methods such as presentations, group talks and activities. Space for men to discuss issues, seek advice and guidance, and express difficult emotions in a safe, empowering setting. Elders joined workshops as peers/mentors.	NR.
Dreaming inside. *Hanley and Marchetti 2020 (Peer‐reviewed journal article)*. *Marchetti and Nicholson 2020 (Peer‐reviewed journal article)*.	Arts‐based prison program. Culturally safe creative writing program to empower and heal Aboriginal and Torres Strait Islander men in prison.	Junee, NSW—MM 5. Junee Correctional Centre.	Aboriginal and Torres Strait Islander men in the prison. 86 men contributed to published work. Age range 20–50+ years.	Workshops in a culturally safe environment, on how to write poems and stories on any topic and how to become published authors. The Elders and Aboriginal tutors encourage the men to talk about their experiences while planning on topics to write about.	The main benefit identified by most (70%) men who were interviewed was the program allowed them to tell their story. Engaging with the program improved well‐being for 33% of interview participants.
MomenTIM—Tomorrows Indigenous Men. *Smith* et al *2020 (Guide/Resource)*. *Movember n.d. (Website)*	Health education, promotion and prevention for young men. Aimed at de‐stigmatising mental health and encouraging personal conversations about mental health.	Moreton Bay, QLD—MM 1. Deception Bay, QLD—MM 1. Mount Isa, QLD—MM 7. Wellington, NSW—MM 5. Predominantly a school‐based program.	12–25‐year‐old Aboriginal and Torres Strait Islander males.	A school‐based program delivering 4 main topics: self‐care, cultural identity, mental health and healthy relationships. A marketing campaign includes a line of fashionable and popular apparel for young Indigenous men to be available through incentivised health checks and program participation.	Indicative success based on the feedback. More positive impacts noted in the family unit and re‐engagement into school.
Camping on Country—Our health, Our Way (Men's movement). *Camping on Country 2021 (Website)*.	Health and culture. Has a focus on wellbeing through cultural connection on country.	Camping on country in 9 locations: Normanton, QLD—MM 7; Kunanurra, WA—MM 7; Borroloola, NT—MM 7; Tennant Creek, NT—MM 7; Kowanyama, QLD—MM 7; Mareeba, QLD—MM 5; Cooktown, QLD—MM 6; Wujal Wujal, QLD—MM 6; Hopevale, QLD—MM 6.	279 participants (Mar 2018‐ July 2021) across all locations.	Health checks on country, participation in smoking cessation workshops, healthy eating and bush tucker and cultural activities. Services: Counselling, commercial grade washing and hygiene services (hot showers).	NR.
A pilot study using a small‐sided games program to modify cardiovascular health in sedentary Indigenous men. *Sampson* et al *2021 (Peer‐reviewed journal article)*.	Exercise program to determine cardiovascular health benefits in Indigenous men following short‐duration small‐sided games.	Illawarra community, NSW—MM 4. Team sport. Setting NR.	14 sedentary Indigenous males.	A modified touch football game consisting of 4 bouts of 5‐minutes duration interspersed with 2 minutes rest.	Middle‐aged Indigenous men can gain cardiovascular health benefits following short bouts of small‐sided game play accumulating in 40‐minutes of exercise each week.
His Tribe Aboriginal‐Designed Empowerment Program. *Gee* et al *2022 (Peer‐reviewed journal article)*.	Empowerment program (pilot). Designed to strengthen mental health, SEWB, community connection, reduce psychological distress and the risk of chronic disease.	Melbourne, VIC—MM 1. Program occurred at a Victorian local Aboriginal community‐controlled health service.	37 Aboriginal and Torres Strait Islander men attended at least one session of the programs.	Sessions included inspirational presentations by Aboriginal speakers and smoking ceremonies. Participants trained together for 1 h, engaging in physical activities (circuits, weights, group competitions and cultural games). Weekend activities included hiking, playing cricket, roller skating, and attending a football game.	No significant increase in aerobic fitness or weight changes for male participants. Data from yarning circles suggested participants experienced a wide range of SEWB benefits from the program.

*Note*: Geo‐locations described using the Modified Monash Model (MM 2019) classification codes, developed by The Australian Government Department of Health and Aged Care: Metropolitan areas (MM 1), Regional centres (MM 2), Large rural towns (MM 3), Medium rural towns (MM 4), Small rural towns (MM 5), Remote communities (MM 6), Very remote communities (MM 7)—(https://www.health.gov.au/resources/apps‐and‐tools/health‐workforce‐locator/app).

Abbreviations: AFL, Australian Football League; AOD, alcohol and other drugs; BBQ, barbeque; BSFC, Broom Saints Football Club; FDV, family domestic violence; kg, kilograms; NJP, Ngarra Jarranounith Place; NR, not reported; NT, Northern Territory; NSW, New South Wales; QLD, Queensland; Remote and Metropolitan Area; RRMA, Rural; SA, South Australia; SEWB, social and emotional wellbeing; VIC, Victoria; WA, Western Australia.

^a^ ‘Koori(e)’ is a demonym for Aboriginal Australians from a region that approximately corresponds to southern New South Wales and Victoria.

^b^ ‘Murri’ is a demonym for Aboriginal Australians of modern‐day Queensland and north‐western New South Wales.

#### 3.2.1 Program scope

Of the 54 programs, *n* = 23 addressed violence (family and sexual) or violent behaviour^33^, ^34^, ^35^, ^36^, ^37^, ^38^, ^39^, ^40^, ^41^, ^42^, ^43^, ^44^, ^45^, ^46^, ^47^, ^48^, ^49^, ^50^; *n* = 7 addressed lifestyle behaviours (e.g., physical activity, nutrition and weight management)^51^, ^52^, ^53^, ^54^, ^55^, ^56^, ^57^; *n* = 6 focused on supporting fathers^44^, ^58^, ^59^, ^60^, ^61^, ^62^, ^63^; *n* = 7 were described as empowerment, social and emotional wellbeing or healing programs,^38^, ^43^, ^44^, ^61^, ^64^, ^65^, ^66^ with one having a particular focus on cultural connection on country^66^; *n* = 5 focused on strengthening cultural identity, including participants' understanding of their role in their family and community^36^, ^37^, ^43^, ^49^, ^67^, ^68^; and *n* = 4 focused on reducing suicide risk.^36^, ^37^, ^40^, ^41^, ^69^, ^70^ Under half of the identified programs (*n* = 23) imposed age restrictions on who could join; *n* = 17 programs were targeted towards adults (aged ≥18 years), and *n* = 6 programs were explicitly targeted towards ‘young’ males (aged 15‐35 years).^53^, ^54^, ^58^, ^63^, ^65^, ^71^ The activities and content within programs varied. Ten programs were explicitly described as ‘men's groups’.^33^, ^34^, ^36^, ^37^, ^40^, ^41^, ^42^, ^43^, ^44^ Cultural strengthening activities were common features of programs: *n* = 6 included art‐based activities such as writing, poetry and other related cultural activities for behaviour change and empowerment^35^, ^44^, ^45^, ^49^, ^60^, ^72^, ^73^; *n* = 12 included camping^33^, ^34^, ^38^, ^43^, ^44^, ^48^, ^61^, ^64^, ^65^, ^66^, ^67^; *n* = 6 programs included fishing and/or hunting as cultural activities.^41^, ^43^, ^44^, ^48^, ^74^

#### 3.2.2 Program geographical location and setting

Programs found in this review were delivered across most, but not all, Australian states and territories. They were New South Wales (NSW) (*n* = 19), Queensland (QLD) (*n* = 15), Northern Territory (NT) (*n* = 11), Victoria (VIC) (*n* = 10), Western Australia (WA) (*n* = 6) and South Australia (SA) (*n* = 3). Programs delivered in specified geographic locations, were in metropolitan (*n* = 17), regional centres (*n* = 4), large rural towns (*n* = 6), medium rural towns (*n* = 4), small rural towns (*n* = 7), remote community (*n* = 8), very remote community (*n* = 9) areas (*n* = 8 not reported). The Mibbinbah^75^ and Camping on Country^66^ programs occurred across multiple jurisdictions, spanning various rural and remote locations. Just over half of included publications (*n* = 28) stipulated the setting where the programs were delivered: *n* = 6 programs were delivered in prison/correctional centre settings,^35^, ^39^, ^44^, ^45^, ^60^, ^72^, ^73^ *n* = 6 programs were delivered in a ‘Men's shed’,^43^, ^44^, ^48^, ^69^, ^75^, ^76^ *n* = 5 programs were delivered at health services,^39^, ^40^, ^41^, ^42^, ^59^, ^77^ *n* = 4 programs occurred at sporting events/training or clubs,^51^, ^53^, ^70^, ^71^ one of which was also delivered at a health clinic,^53^ *n* = 4 programs took place in various community centres,^49^, ^52^, ^54^, ^64^ and *n* = 2 programs were delivered via camping trips ‘on Country’.^38^, ^66^ One program was implemented as a school‐based program.^57^, ^78^

### 3.3 Key Aboriginal and Torres Strait Islander male health program elements

The reporting of key program elements in each source are shown in Table 2. The extent to which the seven researcher‐identified key program elements were reported varied significantly among the included publications. All seven program elements were identified for nine programs,^36^, ^37^, ^40^, ^41^, ^42^, ^45^, ^50^, ^61^, ^67^, ^72^, ^73^, ^77^ six of which had a single source associated with it.^42^, ^45^, ^50^, ^61^, ^67^, ^77^ Conversely, one included source (fact sheet on the Jumna Wal program), only reported on one program element.^64^

**TABLE 2:** Key program elements.

Program Name & Citations	Origins of Program Design	Program Governance	Program Leads / Facilitators	Program Funding Source	Program Duration and Frequency	Program Outcomes and Measures	Program Monitoring and Evaluation
Koori* Men Exploring New Directions Group.^33^, ^34^	There is close collaboration with a local Aboriginal rehabilitation centre.	NR.	1 Aboriginal facilitator.	A small grant from the Commonwealth Department of Primary Industries enabled the set‐up groups co‐facilitated by a team of trained and experienced groupworkers.	Weekly.	Changes in violent behaviour measured through surveys, observation and discussion with a range of stakeholders.	Monitoring and evaluation plan NR.
Well men's check‐ups.^51^	The Nhulunbuy Health Promotion Team.	NR.	Aboriginal health workers and health promotion officers.	NR.	Opportunistic ‐ at football games and training.	Biometric screening, questionnaire on lifestyle habits, stress/distress.	NR.
GutBusters.^52^	The `GutBusters' program was modified by and for local Indigenous men.	NR.	22 male community representatives were trained. Elders, as well as Indigenous male health workers, leaders and advocates.	Grant from Diabetes Australia	Conducted over 1 year.	Men were weighed and measured for height, sitting height (not reported here), waist and hip circumference, or as many of these as was possible or practical at the time of testing.	NR.
Indigenous Young Fathers' Support Group.^58^	The author (Indigenous) and discussions with young Indigenous fathers, mothers and community agencies.	NR.	Facilitators together with participants who led decisions on how the group would operate.	NR.	NR.	NR.	NR.
Sex offender program.^35^	Program originally designed and delivered in Scotland. However, was modified by program developers and Indigenous consultants and offenders for appropriateness and suitability.	Indigenous men's committee.	Indigenous facilitators (Aboriginal and Samoan/Maor).	NR.	NR.	NR.	Ongoing at time of publication. Qualitative, Intervention Research methods.
Yarrabah Men's Health Group.^36^, ^37^	Men in the Yarrabah community acting as role models, in response to wave of youth suicides and other needs of local men.	Gurriny Yealamucka Health Service Governing Committee. Community members also engaged in governance roles.	2 local men employed, (1 full‐time and 1 part‐time) coordinate and support the activities of the Men's Group. Aboriginal community members were trained and employed as facilitators.	2‐year National Suicide Strategy funding (Aus. Govt. Dept. of Health & Aged Care). 1 full‐time position, 1 part‐time position.	Fortnightly, 1‐2 hours.	Improve SEWB, mental health. Participants rated individual performances and behaviours against a set of criteria using a simple scale from 0 (poor) to 10 (excellent). After 2 years, majority of men improved their rating by 4‐5 points. A set of principles designed by participants acted as personal goals to gradually achieve.	PAR approach to enable evaluation process.
The Footy Fitness Project.^53^	Arose from community concerns regarding the health of young Aboriginal men.	NR.	NR.	NR.	Conducted over 2 years.	Assessment included recording demographic details, cardiac risk factors and symptoms, physical examination by a doctor, resting ECG and spirometry, fitness test using an exercise bike, blood and urine samples taken for analysis. A simple standardised data collection form was designed for the assessments.	An evaluation questionnaire sent to community members after completion of program assessments.
Wadja Warriors ‐ healthy weight program.^54^	Initiated by the Wadja Warriors, Woorabinda's football team.	NR.	Facilitators and support people, including an Elder.	NR.	Workshops conducted over 8 months in total.	Screenings for diabetes and other conditions. Measurement of personal fitness at the start and end. Questionnaire at the start and end of program, informed improvement and/or maintenance of healthy lifestyle choices/behaviour.	Mixed methods (scale rating form and questionnaire).
Empowering men to be who we know they are.^38^	Health workers from the Wheatbelt Aboriginal Corporation.	NR.	NR.	NR.	3‐day camp.	NR.	NR.
The Countrymen Program.^39^	Australian Government Initiative.	NR.	The program was redesigned to provide training for Indigenous support workers and counsellors.	Aus. Govt. funded initiative ‐ Partnerships Against Domestic Violence	NR.	NR.	A meta‐evaluation was conducted.
Counselling program for Indigenous men who use violence.^39^	Australian Government Initiative.	NR.	NR.	Aus. Govt. funded initiative ‐ Partnerships Against Domestic Violence	12–14 weeks.	NR.	A meta‐evaluation was conducted.
Ending Family Violence Program.^39^	Australian Government Initiative.	NR.	NR.	Aus. Govt. funded initiative ‐ Partnerships Against Domestic Violence	8 x 2‐hour sessions.	NR.	A meta‐evaluation was conducted.
HomeGround's Koori Men's Outreach Recreation Program.^74^	NR.	NR.	NR.	NR.	Spontaneous.	Re‐creation of the ‘self’ is documented with photographs and narratives.	NR.
Yaba Bimbie Indigenous Men's Support Group.^40^, ^41^	Auspiced by Yarrabah's community‐controlled health service, Gurriny Yealamucka in response to community needs.	A Governing Committee of key community/family members of Yarrabah to be established.	2 facilitators (local men). Later several members of the group were trained as facilitators.	Group operated on a voluntary basis for 2 years. Funding was then received from the National Suicide Prevention Strategy (Aus. Govt. Dept. of Health & Aged Care, 2001‐03) and the National Health and Medical Research Council (2004‐06). *See paper for subsequent funding for other program activities.	Once a week.	Personal growth and development. Participant observations, reflections of Men's group project workers, routinely collected Men's group activity data, some community‐level statistical data, and interviews.	Mixed methods (interviews, activity data). PAR approach to enable evaluation process.
Ma’Ddaimba‐Balas Indigenous Men's Group.^42^	The Mamu Medical Service and the community.	Research program steering committee. In 2003, the Ma’Ddaimba‐Balas Men's Group was incorporated, and a Board was formed.	Men's group leaders. Members of the group employed as research workers to work with men in the local community.	Partnership with Yaba Bimbie Men's Group and University of Queensland/James Cook University researchers. Top‐up funding assistance from the Community Development Employment Program. 2 x full‐time positions. 2 x part‐time research support workers for 3 years.	Once a month.	Questionnaire to Men's group leaders, interviews, collection of group activity data and statistical data on Indigenous representation in the court system. Health screening for sugar, high blood pressure, cholesterol and weight.	Mixed methods (interviews, activity data). PAR approach to enable evaluation process.
Koori Fathering Program.^59^	Health Promotion Unit at Northern Rivers Area Health Service, sought opinions from local Elders, community members and other organisations.	NR.	3 facilitators were local Aboriginal men and fathers, also acting as participants, sharing their experiences and emotions.	Northern Rivers Area Health Service (NSW Govt.) funded and supported all stages of the project.	Once a week x 3 hours. 15‐week course.	Participants’ perceived changes measured via self‐rating scale of 1 (poor) to 10 (great) during sessions, and pre and post program interviews.	Mixed methods (interviews, self‐ratings, surveys).
Rekindling the Spirit Program.^43^, ^44^	Indigenous owned, developed and run initiative.	NR.	Rekindling the Spirit is run and staffed by Indigenous people (e.g., Aboriginal counsellors).	NSW Government's Two Ways Together plan. Also relies on ‘other’ funding bodies to keep it running.	Once a week x 4 hours over 12 weeks. 3‐night camp, twice a year.	NR.	NR.
Yerli Birko (Men's Group).^43^	A collaborative of Nunkuwarrin Yunti ‐ Aboriginal Medical Service SA and the Aboriginal Sobriety Group, building on existing programs and expertise.	Cultural Reference Group, including Elders and other's experienced with working with Indigenous young men from the health and social services.	NR.	Annual cost of running the group (including wages for workers) is approximately $65,000, mostly provided by Nunkuwarrin Yunti of SA (ACCHO). Also, reliant on volunteers to help run program activities.	NR.	NR.	NR.
Spirited Men.^43^	Initiative of the Lower Murray Nungas Club and Kalparrin Community Incorporated.	NR.	3 of the facilitators are also active members of the group.	Family Violence Regional Activities Program (Commonwealth Department of Families, Communities Services and Indigenous Affairs). Approximately $500,000 was secured to fund the continuance of the group for three years. 1 full‐time and 3 part‐time employees.	Once a week.	NR.	NR.
Tau Nagaraldi ‐ Anger management and family violence program.^43^	Tau Ngaraldi grew from the Spirited Men group (see above) and developed by Mack Hayes (facilitator).	NR.	Facilitated by Indigenous men.	NR.	NR.	NR.	NR.
The Mount Isa Murri** Court – Men's support group.^43^	Community generated.	Inbuilt, authorised governance by way of involvement (advising/assisting) of Indigenous Elders or respected persons in the court process.	Facilitated by a local man. Elders involved as mentors/role models.	NR.	Once a week.	NR.	NR.
The Koori Cognitive Skills Program.^45^	Corrections Victoria engaged Robin Jones, Graham Atkinson and Tania Jones (Indigenous) to advise on modifying the mainstream Cognitive Skills Program to be responsive to Koori offenders and prisoners in Victoria.	A 10‐member Reference Group, consisting of seven Indigenous members (6 Aboriginal and 1 Maori), and 3 non‐Indigenous members.	3 Koori facilitators (respected Elders) co‐led the program with non‐Indigenous psychologists. At 2 sites Program Support Officers were Indigenous members of prison staff.	Commissioned by Corrections Victoria (VIC. Govt.) in 2003.	30 x 2‐hour sessions.	Combination of psychometric assessment and videotaped role‐play data (observation). The Problem‐Solving Inventory (questionnaire) was administered at the start and end of the program.	Mixed methods. Process and impact evaluation.
The Miller Aboriginal Men's Group Bike Fleet.^55^	Collaboration between the Miller Community Centre Aboriginal men's group, Sydney Southwest Area Health Service Health Promotion Service and the 'Cycling Connecting Communities' program	NR.	Men's group coordinator, cycling coach, and health promotion managers.	Funded by NSW Health (Govt.). 6 bikes provided by the Miller Hub Community Centre.	NR.	Program and course participation.	Yes. Monitoring and evaluation plan NR.
“Mibbinbah” Indigenous Men's Shed.^75^	Rick Hayes, an American senior lecturer at La Trobe University was key to development of the program.	NR.	Local Aboriginal and Torres Strait Islander Male project associates.	The pilot phase, jointly funded by the Chronic Disease (Conditions) Program of the Cooperative Centre for Aboriginal Health and Beyond Blue.	NR.	NR.	NR.
Brothers inside.^60^	Craig Hammond (Family Action Centre, The University of Newcastle) sought input from Aboriginal staff at the Prison.	NR.	Aboriginal teacher/facilitator.	NR.	Once a week x 4 hours, over 8 weeks.	NR.	NR.
The Shed in Mount Druitt.^69^	Founded as a partnership between Western Sydney University, Dr Bob Morgan (Aboriginal scholar), the Holy Family Church and the Mt Druitt Aboriginal community.	NR.	Employment of 2 Aboriginal workers. The shed takes guidance from Aboriginal males. The men identify what services should visit the Shed (e.g., Aboriginal health workers).	Funded by a grant from the Federal Government, Department of Health and Ageing, under their Suicide Prevention Program (Aus. Govt. Dept. of Health & Aged Care). Employment 2 x Aboriginal workers.	NR.	NR.	NR.
Alive and Kicking Goals!^70^	Initiated by members of the Broome Saints Football Club (predominantly Indigenous).	Youth sub‐committee made up of players at the football club who were empowered to be the decision makers of the project.	Young members (Aboriginal) of the football club and the youth sub‐committee, were trained to be suicide prevention mentors and Peer Educators.	Healthway WA, Mens Outreach Service, FaHCSIA, headspace Kimberley, Rio Tinto, Kinway and Anglicare WA funding supported the 12‐month pilot.	Once a week, over 1 year.	NR.	NR.
Jumna Wal ‐ Creating Better Pathways for Men.^64^	NR.	NR.	NR.	NR.	Over 9 weeks.	NR.	NR.
Tweed Yarn Up Group.^46^	Designed by Stuart Anderson (from the Lismore Men and Family Centre) and adapted and facilitated by Greg Telford (from Rekindling the Spirit, Limited).	NR.	Aboriginal group/workshop facilitator.	Young Women's Christian Association, Communities for Children program, Tweed Valley Early Childhood Intervention Service, On Track Community Programs’ Bunyarah‐ga Drug and Alcohol program, Tweed Aboriginal Cooperative Society and New Horizon provided the funding, space and other resources.	Once a week x 3 hours, over 14 weeks.	Records of participant attendance, engagement, feedback and observational notes taken by facilitators in each session.	Weekly check‐ins (discussions) with group took place in each session for feedback.
The Koorie* Men's Health Day.^56^	Requested by Elders from the region and was recognised as a need of the community.	Steering group included professionals from the local mental health service and 4 key individuals from the local Aboriginal community.	Aboriginal health worker.	Beyond Blue Strategic Research Grant Program.	1‐day event.	Complete medical examination, point of care testing. Psychological distress measured using the Kessler 10 questionnaire (K‐10).	NR.
Gatharr Weyebe Banabe Program.^47^	Adapted to suit Aboriginal and Torres Strait Islander men. Changes driven by lived experiences of facilitators, listening to families, and the guidance of family Elders.	Engagement with and accountability to local Traditional Owners and recognised Elders, who also continually assessed for cultural appropriateness.	Aboriginal and Torres Strait Islander male facilitators.	NR.	Phase 1: one week ‐ 3 months. Phase 2: retreat, typically 4 days. Phase 3: eight weeks mandated (often continues for 5 months).	NR.	NR.
The sport and active recreation programs in an Indigenous men's shed.^76^	The coordinator of the Shed and one of the Shed members.	NR.	Men's shed coordinator.	NR.	10 weeks. Healthy eating program is 6 months.	Semi‐structured interviews and yarning circles.	NR.
Maambart Maam For Maali Moort Wellbeing Pilot Program.^61^	Aboriginal designed, led and delivered wellbeing program.	Executive Committee, Expert Advisory Groups and a Community reference group.	Aboriginal led, and culturally secure.	Funded by BeyondBlue with donations from The Movember Foundation. Other funders included Healthway and the local government authority, City of Swan, WA.	15 activities over 3‐month period.	Interviews and post program evaluations, feedback form and questionnaires. 3 culturally appropriate measures: Indigenous Racial Identity and Self‐esteem for Aboriginal Adults (IRISE_A), Perceptions of Aboriginal People and Culture for non‐Aboriginal Adults, and Aboriginal and Islander Mental Health Initiative (AIMhi) Brief Wellbeing Screener	Mixed methods (interviews, scale ratings, surveys). PAR approach to enable evaluation process. Quality of program delivery was also continually monitored.
A 12‐week sports‐based exercise programme.^80^	NR.	NR.	NR.	Research Grants from the University of Technology Sydney, Faculty of Business and Charles Sturt University, Faculty of Education.	2–3 days a week, over 12 weeks.	Pre‐ and post‐intervention testing included: anthropometry, peak aerobic capacity, fasting blood chemistry of inflammatory cytokines, adiponectin, leptin, cholesterol, glucose, insulin and C‐peptide.	NR.
Our Men, Our Healing. Wurrumiyanga – Tiwi Men's Healing Program.^48^, ^67^	Co‐designed by local men and leaders with support from the Healing Foundation and Catholic Care NT.	Knowledge Circle of 7 Indigenous men and 1 man who identifies as Papa New Guinean.	A local Aboriginal man as the cultural group facilitator. Cultural knowledge and mentorship by Elders.	$600,000 (split across all 3, Our Men, Our Healing’ programs) from the Northern Territory Department of Children and Families.	NR.	NR.	Mixed methods. Input from participants, service providers, stakeholders and community members.
Our Men, Our Healing. Gurrutu Raypirri Men's Healing Project.^48^	Co‐designed by local men, leaders and key representatives from local stakeholder groups.	Knowledge Circle of 7 Indigenous men and 1 man who identifies as Papa New Guinean.	Coordinator of the program is a local Indigenous man.	$600,000 (split across all 3, Our Men, Our Healing’ programs) from the Northern Territory Department of Children and Families.	NR.	NR.	Mixed methods. Input from participants, service providers, stakeholders and community members.
Our Men, Our Healing. Ngukurr Men's Cultural Healing Program.^48^	Co‐designed by local men, leaders and key representatives from local stakeholder groups.	Knowledge Circle of 7 Indigenous men and 1 man who identifies as Papa New Guinean.	Auspiced by Sunrise Health Service Aboriginal Corporation.	$600,000 (split across all 3, Our Men, Our Healing’ programs) from the Northern Territory Department of Children and Families.	NR.	NR.	Mixed methods. Input from participants, service providers, stakeholders and community members.
Quop Maaman: Aboriginal Fathering Project.^62^, ^67^	Developed by Moodjar Consultancy (Indigenous owned company) and designed with Indigenous scholars and local men.	Project Steering group.	Facilitators are Aboriginal, senior, cultural men.	NR.	NR.	NR.	Mixed methods (feedback discussions, questions sheets, scale ratings).
The Blue Mountains Aboriginal Men and Youth Program.^65^	Aboriginal Elders and service providers in Blue Mountains (BM) areas, contacted the City Council of BM. Developed in response the community needs.	The initial steering committee for the AMYP was predominately non‐Indigenous females.	A senior Aboriginal male (local to the area). Every activity delivered in the AMYP was supported by Elders.	NSW Department of Family and Community Services (defunct) and Community Builders Fixed‐term Funding Program. 3 years and extended another year.	Monthly meetings, 1‐3 hours. Full day workshops and forums. Boxing program, 3 x 1‐hour sessions per week at a total of 48 sessions, over 16 weeks.	NR.	Mixed methods (interviews, activity data).
Right Tracks Program.^71^	A local Indigenous man developed the program in response to community needs.	NR.	Coach and founder of the program (Indigenous) Influential males in the community (footballers), and stakeholders.	NR.	Full time program 12 months of the year, 7 days a week. Structured curriculum.	NR.	NR.
Stayin' on Track.^63^	Aboriginal mentors and researchers. Community support from ACCHS and local community.	NR.	20 young Aboriginal fathers recruited as co‐investigators to develop resources. Discussions were guided by 2 Aboriginal male mentors.	Funding support from The Young and Well Cooperative Research Centre and The University of Newcastle.	Ongoing. Internet support resources, always accessible.	Web tracking, phone messages) and Mood Tracker messages using a 5‐point scale ranging from high to low: solid, deadly, OK, mad or low. Appropriate and relevant referrals suggested if required.	Mixed methods (yarning, web tracking). PAR approach to enable evaluation process.
Illawarra Koori Men's Support Group (IKMSG).^79^	Aboriginal men (members) living in the Illawarra area with Aboriginal Elders at its helm and supported by the local community.	Board of Directors consisting of local Aboriginal men and respected Elders who have built credibility and history within the Illawarra community.	The IKMSG is led by a strong group of Aboriginal Elders. Many Aboriginal men employed in liaison roles across various government departments have engaged with the IKMSG.	NR.	Once a week.	NR.	Mixed methods (interviews, activity data, survey, social network analysis). Indigenous research approach.
Strong Men Strong Communities ‐ Men's healing project.^67^	Co‐designed program including local Indigenous leaders and representatives from each of the 7 Darwin Town Communities.	A Reference Group with Indigenous representatives from the community.	Project Officer.	Funding totalling $300,000 from the Commonwealth Department of Indigenous Affairs (defunct) was split between the Healing Foundation and Darwin Aboriginal & Islander Women's Shelter Indigenous Men's Service.	Once a week.	Interviews, participant observations and anecdotal stories from men in the program of improvements in levels of alcohol and drug use, family violence etc.	Mixed methods (interviews, participant observation, document analysis).
The Indigenous Men's Service.^44^	Co‐design process informed by a knowledge circle of nationally recognised Aboriginal and Torres Strait Islander violence prevention experts.	NR.	Indigenous male worker provides family violence counselling, mentoring and group work via outreach.	NR.	NR.	NR.	NR.
Dardi Munwurro's men's healing programs: Strong Spirit Program.^44^	NR.	NR.	Delivered by 2 counsellors.	NR.	Strong Spirit: 12 fortnightly group sessions. 3‐day camp. Also adapted to 8‐day intensive program in correctional facilities.	NR.	NR.
Dardi Munwurro's men's healing programs: The Men's Healing and Behaviour Change program.^49^	Dardi Munwurro is a grassroots Aboriginal community organisation. Guidance and wisdom of Elders.	NR.	2 facilitators supported by community Elders.	NR.	Once a week group session in urban area, fortnightly in wider metropolitan and a 40‐hour, weeklong prison program.	An outcomes tool using a scale from 0 to 10 to measure change in emotional intelligence, social and emotional wellbeing, health, spirit, responsibility, relationships, and culture. Pre and post program data collected on, misuse of alcohol and other drugs, employment, accommodation, justice, episodes of violence.	Mixed methods. Cost‐benefit analysis.
Dardi Munwurro's men's healing programs: Ngarra Jarranounith Place (NJP).^49^	Dardi Munwurro is a grassroots Aboriginal community organisation. Guidance and wisdom of Elders.	The program is overseen by a clinical governance group that includes an Aboriginal psychologist and other professionals.	NR.	NR.	16‐weeks.	An outcomes tool using a scale from 0 to 10 to measure change in emotional intelligence, social and emotional wellbeing, health, spirit, responsibility, relationships, and culture. Pre and post program data collected on, misuse of alcohol and other drugs, employment, accommodation, justice, episodes of violence.	Mixed methods. Cost‐benefit analysis.
Breakthrough Violence Program ‐ Changing Minds and Saving Lives.^50^	Creating a Safe and Supportive Environment (CASSE) and the Central Australian Aboriginal Congress.	The Male Leadership Group, made up of Aboriginal males from the Alice Springs community.	A cultural male facilitator alongside senior social workers, psychologists, and a therapist.	Commissioned by the 2 organisations in partnership, (CASSE) and the Central Australian Aboriginal Congress in 2015.	Once a week x 2 hours, over 15 weeks.	Pre and post measures: The Growth and Empowerment Measure, Kessler 6 Distress Scale including two new items assessing angry and happy feelings, and interviews.	PAR approach. Empowerment Research Program.
Code 4 Life: Arraty Angka ‐ Straight Talk.^68^	Designed by Program Manager (Indigenous man).	Board of Directors, which includes Indigenous individuals.	Workshops are facilitated by Indigenous staff is overseen by Aboriginal Elders and senior community leaders.	NR.	NR.	NR.	NR.
Dreaming inside.^72^, ^73^	Conceived by a Wadi Wadi Elder, Dr Barbara Nicholson. The name ‘Dreaming Inside’ was coined by men participating in the first workshop.	Advisory Group made up of key stakeholders, two of whom were Aboriginal.	Facilitated by Elders (tutors) from the Black Wallaby Writers (South Coast Writers Centre).	Funded by a Global Challenges grant from the University of Wollongong, and Australian Research Council Future Fellowship grant.	5 workshops, over 3 days, twice a year.	Participant interviews and feedback forms.	Mixed methods (interviews, feedback forms; open and closed text responses). A small‐scale outcome evaluation.
MomenTIM‐ Tomorrows Indigenous Men.^57^, ^78^	The Institute for Urban Indigenous Health (IUIH). Culturally appropriate program based on input from local Aboriginal stakeholders.	Governance Committee. Members include the IUIH CEO, CEOs from 3 Aboriginal Medical Services, IUIH Clinical Services Director, Uni. of QLD Research Officers, MomenTIM Regional Coordinator and IUIH Communications Team Leader.	Clinical and non‐clinical services in an integrated, whole of community approach.	The Movember Foundation. $2,675,800 funded from December 2014 – to date.	NR.	NR.	NR.
Camping on Country ‐ Our health, Our Way (Men's movement).^66^	Indigenous actor/comedian/presenter and Elders.	NR.	NR.	The Australian Government Department of Health (and Aged Care).	5‐day camps.	Participant impact statements. Program activity data.	NR.
A pilot study using a small‐sided games program to modify cardiovascular health in sedentary Indigenous men.^81^	NR.	NR.	NR.	Supported by a Vanguard Grant (Award) from the National Heart Foundation of Australia.	Twice a week, over 9 weeks.	Waist and hip circumferences, height, total body mass (kg), fat (%), fat free mass (kg), muscle mass (kg), resting heart rate (bpm), systolic blood pressure (mmHg), cholesterol and high‐density lipid concentrations, waist‐hip ratios, body mass index, heart rate variability, metabolic age and Framingham risk calculated before and after the program.	NR.
His Tribe Aboriginal‐Designed Empowerment Program.^77^	A team of people from the Victorian Aboriginal Health Service. Aboriginal voices privileged throughout the design, implementation, and evaluation of the programs.	Reference group that included Aboriginal Elders and community leaders.	Included Indigenous program leads /researchers. Speakers at sessions were well‐known local Aboriginal community leaders, or national Aboriginal figures.	Victorian Department of Health and Human Services.	Once a week x 2.5 hours, over 12 weeks.	Aboriginal Resilience and Recovery Questionnaire using a 5‐point Likert scale response with ratings from 1‐5 and includes strength‐based constructs and sub‐scales. Kessler Psychological Distress Scale (K10). Body weight and aerobic capacity measures pre and post program. 6‐month follow up.	Mixed methods (questionnaire yarning, semi‐structured questions). Participants engaged in evaluation component, led by Aboriginal researchers.

*Note*: Grey shade indicates involvement/input from Aboriginal and Torres Strait Islander individuals/community.

Abbreviations: ACCHO, Aboriginal Community Controlled Health Organisation; ACCHS, Aboriginal Community Controlled Health Service; AFL, Australian Football League; CEO, Chief Executive Officer; ECG, electrocardiography; FaHCSIA, former Australian Department of Families, Housing, Community Services and Indigenous Affairs; IUIH, Institute for Urban Indigenous Health; kg, kilograms; mmHg, blood pressure millimetres of mercury; NJP, Ngarra Jarranounith Place; NR, not reported; NSW, New South Wales; NT, Northern Territory; PAR, participatory action research; QLD, Queensland; SA, South Australia; SEWB, social and emotional wellbeing; VIC, Victoria; WA, Western Australia.

^a^ ‘Koori(e)’ is a demonym for Aboriginal Australians from a region that approximately corresponds to southern New South Wales and Victoria.

^b^ ‘Murri’ is a demonym for Aboriginal Australians of modern‐day Queensland and north‐western New South Wales.

The type of source influenced the level of detail reported, for example, peer‐reviewed publications tended to report on a greater number of key program elements compared to grey literature publications. Four reports exploring more than one program, had inconsistencies between what elements were reported for each program^39^, ^43^, ^44^, ^49^ while another consistently reported the same elements for the five programs described.^48^ Evaluation reports describing a single program tended to report on six to seven elements,^42^, ^46^, ^59^, ^61^, ^65^ with one only reporting four.^62^

#### 3.3.1 Program origin

The origin of a program was the most reported program element, reported for 49 of 54 programs. Of these, *n* = 44 programs were described to have been instigated or co‐designed by Aboriginal and Torres Strait Islander individuals/community to suit cultural and community needs.^35^, ^36^, ^37^, ^38^, ^40^, ^41^, ^42^, ^43^, ^44^, ^45^, ^46^, ^47^, ^48^, ^49^, ^50^, ^52^, ^53^, ^54^, ^55^, ^56^, ^57^, ^58^, ^59^, ^60^, ^61^, ^62^, ^63^, ^65^, ^66^, ^67^, ^68^, ^69^, ^70^, ^71^, ^72^, ^73^, ^76^, ^77^, ^79^ Two programs delivered by the Dardi Munwurro, Aboriginal‐controlled organisation,^49^ the Maambart Maam for Maali Moort Wellbeing Pilot Program^61^ and the Quop Maaman: Aboriginal Fathering program^44^, ^62^ are programs that were identified as being entirely conceived, designed and led by Aboriginal and Torres Strait Islander people and communities. Likewise, the Rekindling the Spirit program is an Indigenous owned and run initiative,^43^, ^44^ and the Alive and Kicking Goals program is described as ‘wholly owned’ by the community.^70^ Six pre‐existing programs were specifically adapted to suit delivery to the Aboriginal and Torres Strait Islander males, addressing their respective needs.^35^, ^43^, ^45^, ^46^, ^47^, ^52^ Individuals, Elders and/or community members, initiated *n* = 10 programs due to their concerns about male health and wellbeing,^36^, ^37^, ^40^, ^41^, ^43^, ^53^, ^54^, ^55^, ^56^, ^58^, ^65^, ^70^, ^71^, ^79^ four of which were in response to high suicide rates.^36^, ^37^, ^40^, ^41^, ^65^, ^70^

#### 3.3.2 Program governance

Program governance strategies were reported for *n* = 24 programs, and where specified, these commonly included steering committees (*n* = 6), advisory and/or community reference groups (*n* = 4).^35^, ^36^, ^37^, ^40^, ^41^, ^42^, ^43^, ^45^, ^47^, ^48^, ^49^, ^50^, ^56^, ^61^, ^62^, ^65^, ^67^, ^68^, ^70^, ^72^, ^77^, ^78^, ^79^ Program governance involving Aboriginal and Torres Strait Islander people was reported for *n* = 22 programs.^35^, ^36^, ^37^, ^40^, ^41^, ^43^, ^45^, ^47^, ^48^, ^49^, ^50^, ^56^, ^61^, ^67^, ^68^, ^70^, ^72^, ^73^, ^77^, ^78^, ^79^ A variety of governance strategies were reported, highlighting the different approaches taken by those in governance roles who aimed to work with community members and stakeholders to guide and direct programs. The Our Men, Our Healing programs were informed and overseen by eight community members and/or stakeholders who engaged in ‘knowledge circles’.^48^ The Codes 4 Life program^68^ utilised a Board of Directors comprising community Elders and members. The Maambart Maam for Maali Moort Wellbeing Pilot Program indicated using several governance measures – an Executive Committee, Expert Advisory Groups and a Community Reference Group.^61^

#### 3.3.3 Program leads/facilitators

Program leads/facilitators were reported for 43 programs, and of these, 38 appointed Aboriginal and Torres Strait Islander program leads.^33^, ^34^, ^35^, ^36^, ^37^, ^39^, ^40^, ^41^, ^42^, ^43^, ^44^, ^45^, ^46^, ^47^, ^48^, ^49^, ^50^, ^51^, ^52^, ^56^, ^58^, ^59^, ^61^, ^62^, ^63^, ^65^, ^68^, ^69^, ^70^, ^71^, ^72^, ^73^, ^75^, ^77^, ^79^ 13 of these were identified as being local individuals/community members.^36^, ^37^, ^40^, ^41^, ^42^, ^43^, ^48^, ^52^, ^59^, ^65^, ^70^, ^71^ Members/participants of four men's groups also acted as facilitators of the program.^40^, ^42^, ^43^, ^59^ Six programs included training for Aboriginal and Torres Strait Islander participants to become program leads/facilitators,^37^, ^39^, ^40^, ^52^, ^63^, ^70^ such as the Alive and Kicking Goals program, which saw 16 participants of the program become trained as peer educators in suicide prevention.^70^ Ten programs were described as specifically including support, mentorship or facilitation by elders.^43^, ^44^, ^45^, ^49^, ^52^, ^54^, ^65^, ^68^, ^72^, ^73^, ^79^

#### 3.3.4 Program funding

Funding source(s) were stated for 34 programs. Single source funding ranged from Australian Federal and State Government departments (*n* = 15); non‐government, not‐for‐profit organisations/foundations and charities (*n* = 6); research grants and/or universities (*n* = 3) and one provided by an Aboriginal Community Controlled Health Organisation (ACCHO). Ten programs were co‐funded through multiple sources, such as government, non‐government organisations/foundations, research grants, large companies (e.g., mining company), local councils and/or health services.^33^, ^40^, ^41^, ^42^, ^46^, ^50^, ^61^, ^63^, ^70^, ^75^ Funding amount was indicated for seven programs,^43^, ^44^, ^48^, ^67^, ^78^ length of funding provision for five^36^, ^42^, ^43^, ^65^ and seven publications specified how the funds were used, such as employment of program delivery personnel,^33^, ^36^, ^42^, ^43^, ^59^, ^69^ with one adding that volunteer services are essential to help run program activities for the Yerli Birko's men's group.^43^

#### 3.3.5 Program duration and frequency

Program duration and frequency were reported for 39 programs. Program durations were highly varied but typically ranged between 8 and 16 weeks (*n* = 13).^39^, ^44^, ^46^, ^47^, ^49^, ^50^, ^59^, ^60^, ^61^, ^64^, ^65^, ^76^, ^77^, ^80^, ^81^ Programs focused on health behaviour change were typically longer in duration, particularly those programs with a research focus, for example, the GutBusters weight loss program^52^ was 1 year in duration and evaluated program effectiveness for weight loss among Torres Strait Islander males. The Stayin’ on Track program utilises online and mobile phone resources to support young fathers and is the only program that was designed to be accessible on an ongoing basis.^63^ The frequency of sessions within programs was also varied but often included weekly activities (*n* = 14).^33^, ^34^, ^41^, ^43^, ^46^, ^50^, ^59^, ^60^, ^65^, ^67^, ^70^, ^77^, ^79^, ^80^, ^81^ The most infrequent programs were in the setting of events, such as the annual Koorie Men's Health Day, a one‐day sports event program focused on health screening.^56^

#### 3.3.6 Program outcomes and measures

Program outcome measures were reported for *n* = 26 programs.^33^, ^37^, ^41^, ^42^, ^45^, ^46^, ^49^, ^50^, ^51^, ^52^, ^53^, ^54^, ^55^, ^56^, ^59^, ^61^, ^63^, ^66^, ^67^, ^72^, ^73^, ^74^, ^76^, ^77^, ^80^, ^81^ Two publications describing the Yarrabah's Men's Health Group explicitly stated that the participants contributed to determining a set of program outcomes and their measures by developing a set of principles acting as goals to which they aspired to achieve gradually.^36^, ^37^ Likewise, the His Tribe Empowerment Program used the Aboriginal Resilience and Recovery Questionnaire designed by the participating Aboriginal health service,^77^ and the Stayin’ on Track fathering program used mood tracking, scale ratings and internet resources designed and endorsed by young fathers who were co‐investigators in the pilot design.^63^ The Maambart Maam for Maali Moort Wellbeing Pilot Program used a questionnaire designed for Aboriginal participants to measure the perception of racial identity and self‐esteem (I‐RISE: A).^61^ However, in other literature the co‐designing of outcome measures was unclear beyond acknowledging co‐designing of the program and/or using Participatory Action Research (PAR) methods. Outcomes measures included biometric (*n* = 9) (e.g., weight, waist circumference and other chronic disease indicators)^42^, ^51^, ^52^, ^53^, ^54^, ^56^, ^77^, ^80^, ^81^; health behaviours (*n* = 5) (e.g., physical activity, fruit and vegetable consumption, level of alcohol/drug use)^49^, ^51^, ^52^, ^54^, ^67^ knowledge acquisition (*n* = 3) (e.g., cultural knowledge and identity)^49^, ^61^, ^73^; skills (*n* = 4) (e.g., practical parenting and life skills)^45^, ^46^, ^59^, ^67^; and social relationships and wellbeing (*n* = 14) (e.g., social connectivity, supports, social and emotional wellbeing, distress, growth and empowerment).^33^, ^37^, ^41^, ^42^, ^46^, ^49^, ^59^, ^61^, ^63^, ^67^, ^74^, ^76^, ^77^

These were typically collected at a single time point (*n* = 6),^51^, ^56^, ^61^, ^72^, ^73^, ^76^ regularly (*n* = 6),^49^, ^50^, ^52^, ^59^, ^63^ or pre‐post program (*n* = 8).^45^, ^50^, ^53^, ^54^, ^59^, ^77^, ^81^ Furthermore, publications also reported program reach and participant characteristics, attendance numbers, engagement with program online resources (via web tracking) as key outcomes (*n* = 4).^46^, ^55^, ^63^, ^66^ Only one program, the GutBusters program, included a long‐term follow‐up of 12 months.^52^ Of the 26 programs that reported outcome measures, *n* = 23 specified what methods were used, and *n* = 18 used more than one method. The most frequently reported modes of measurement were surveys/questionnaires administered to participant and/or delivery personnel and other self‐reporting methods (*n* = 14).^33^, ^37^, ^42^, ^45^, ^49^, ^51^, ^54^, ^56^, ^59^, ^61^, ^63^, ^77^ Other measures reported were participant observation (*n* = 4)^41^, ^45^, ^46^, ^67^ and interviews (*n* = 8).^41^, ^42^, ^50^, ^59^, ^61^, ^67^, ^72^, ^73^, ^76^

#### 3.3.7 Program monitoring and evaluation

Program evaluations were reported for *n* = 28 programs.^35^, ^37^, ^39^, ^40^, ^41^, ^42^, ^45^, ^46^, ^48^, ^49^, ^50^, ^53^, ^54^, ^55^, ^59^, ^61^, ^62^, ^63^, ^65^, ^67^, ^72^, ^73^, ^77^ Of these, most were published as evaluation reports (*n* = 20),^39^, ^41^, ^42^, ^46^, ^48^, ^49^, ^50^, ^59^, ^61^, ^62^, ^63^, ^65^, ^67^, ^72^, ^79^ commonly in grey literature (*n* = 18).^39^, ^41^, ^42^, ^46^, ^48^, ^49^, ^50^, ^59^, ^61^, ^62^, ^65^, ^67^, ^79^ Evaluation designs for *n* = 4 programs were summative and explored program impacts, such as health and social benefits to participants and the local community.^49^, ^65^, ^67^, ^72^ Two of these also included a cost–benefit analysis and recommendations to sustain the program.^49^, ^65^ Nine programs included a process and summative evaluation.^44^, ^45^, ^46^, ^48^, ^59^, ^61^, ^62^, ^77^ The PAR method was explicitly reported for *n* = 6 program evaluations.^36^, ^37^, ^41^, ^42^, ^50^, ^61^, ^63^ Mixed methods (qualitative and quantitative) and triangulation of data and/or sources were used in *n* = 17 evaluations.^41^, ^42^, ^44^, ^45^, ^48^, ^49^, ^54^, ^59^, ^61^, ^62^, ^63^, ^65^, ^72^, ^77^, ^79^ Five evaluations employed qualitative methods to explore participants perspectives and experiences of the program.^35^, ^46^, ^50^, ^53^, ^67^ Evaluators using PAR methods tended to report a greater diversity of stakeholders involved in the evaluation, such as program participants, Elders, health service staff, and community members.^44^, ^45^, ^46^, ^48^, ^59^, ^61^, ^62^, ^77^

## 4 DISCUSSION

### 4.1 Summary

This review synthesised the literature published on health and wellbeing programs designed for, and delivered to, Aboriginal and Torres Strait Islander males. Only 54 programs were identified, despite not applying any date limiters to the literature search. A small fraction of these (*n* = 6) were published in the last 6 years, illustrating the continuing lack of investment in Aboriginal and Torres Strait Islander male health and the dearth of culturally informed evidence. The included articles lacked consistency in reporting across the program elements we sought to extract, especially outcome measures. Only four identified programs explicitly stated they were evaluated by outcome measures determined in collaboration with Aboriginal and Torres Strait Islander people engaging in the program. Further guidance for health services around how culturally appropriate outcome measures are best developed and implemented can support the evidence base for Aboriginal and Torres Strait Islander male health and wellbeing programs.

### 4.2 Comparison with existing literature

This study is the first to identify the published literature on Aboriginal and Torres Strait Islander male health and wellbeing programs in Australia. The programs exhibited a broad scope and purpose, implemented across various settings throughout Australian states and territories. These studies align with a recent study that collated Aboriginal and Torres Strait Islander chronic disease risk reduction programs.^16^ This diversity demonstrates the versatility and adaptability of Aboriginal and Torres Strait Islander male health and wellbeing programs, provided they are locally and culturally relevant, and guided by the input and needs of participants.^82^, ^83^

Notably, many included programs had an explicit focus on supporting cultural identity and connection among males, two domains which are linked to improved social and emotional wellbeing.^84^ Embedding cultural activities, principles and values in Aboriginal and Torres Strait Islander male health and wellbeing programs aligns with Indigenous conceptions of health and wellbeing.^20^, ^85^ In wider literature, a scoping review of programs and services targeting the social and emotional wellbeing of Aboriginal and Torres Strait Islander young people outlined key principles of such programs. These principles are strongly tied to cultural activities, principles and values, such as the right to self‐determination, recognition of human rights, the recognition of centrality of kinship, and the impact of racism and stigma.^57^, ^86^ Understanding and further exploring the cultural activities, principles and values in the context of Aboriginal and Torres Strait Islander male health and wellbeing programs can support national decolonisation efforts and promote the health and wellbeing of Aboriginal and Torres Strait Islander males. However, we note again that there is currently a disjunct or gap with respect to consistency in the way of assessment or evaluation across programs (key elements; 6. Outcomes and Measures, and 7. Monitoring and Evaluation). Social and emotional wellbeing factors that include cultural determinants of wellbeing, are important and valued by Aboriginal and Torres Strait Islander men. Thought leaders from across Australia gathered in a Northern Territory Think Tank in 2016 and discussed Indigenous male research strategies. They emphasised the crucial need for comprehensive evidence on Aboriginal and Torres Strait Islander men's health programs, their locations, settings and models of care, including what works and why.^87^ This review highlights the importance of reporting on the seven key elements essential to Aboriginal and Torres Strait Islander men's health programs, serving as a foundation for consistent and culturally relevant reporting, to develop a cohesive evidence base. Establishing a set of criteria to inform a global assessment has been implemented with success in other fields, for example the National Drug Research Institute developed elements of best practice to evaluate Indigenous drug and alcohol projects.^88^ In the present review, only six programs published information on all seven elements we sought to extract. Programs with more than one associated publication tended to offer more comprehensive reporting, revealing a high degree of inconsistency across the included publications. In some cases, no input by Aboriginal and Torres Strait Islander people in program design, leadership and facilitation and governance was reported. It is unclear if their input was present and simply not reported, or if it was there was no input at all. The lack of Aboriginal and Torres Strait Islander involvement poses a risk to the cultural safety of program participants. This highlights the need to establish and implement a reporting checklist of these key elements, to increase the consistency and coherency across the body of literature. To advance this field, future research should prioritise transparent and consistent reporting of Aboriginal and Torres Strait Islander male health and wellbeing programs.

### 4.3 Strengths and limitations

This scoping review was led by a Torres Strait Islander man, Associate Professor of Aboriginal and Torres Strait Islander male health and wellbeing (KC). His leadership is a strength due to his academic expertise and extensive networks in the Aboriginal and Torres Strait Islander male health sector, as well as having a deep understanding of the cultural context and guidance of the cross‐cultural research team. Additionally, the input and advice from a further two Torres Strait Islander, and three Aboriginal academic researchers and professionals (co‐authors) ensured cultural perspectives were upheld throughout each stage of the review. The review used broad inclusion criteria, which led to the identification of programs across a variety of settings and locations. A future systematic review expanding on this topic should widen the search strategy and include a greater range of grey literature sources, including websites, and other unpublished work. Regardless, the publications do not include all programs and activities in Australia. Eight publications were not accessible despite multiple efforts of contacting government departments, an author and extensive searches on the internet and databases. The authors acknowledge that there may be programs that have never been published, given the constraints on research, evaluation and publication resources, especially in the settings where programs were identified (e.g., PHCSs, correctional facilities and sporting clubs). Studies included for review were specific to Aboriginal and Torres Strait Islander men's health, however, editorials, perspective, expert opinion, policy, and discussion papers were not considered which may have included Aboriginal and Torres Strait Islander men's programs more generally. This review did not aim to appraise the methodological quality, assess the risk of bias, or analyse program outcomes. Consequently, the effectiveness of the identified programs and the quality and credibility of the included articles remain unknown. The heterogeneity in publication types may indicate poor overall quality, as less than half (45%) of the publications were peer‐reviewed, although this reflects the current evidence base about Aboriginal and Torres Strait Islander male health and wellbeing programs in Australia.

### 4.4 Implications for research, policy and practice

Findings illustrated the considerable diversity across the design, delivery, evaluation and reporting of Aboriginal and Torres Strait Islander male health and wellbeing programs, and this highlights the need for further guidance on best practice in these areas. Future research should advance the conceptualisation of Aboriginal and Torres Strait Islander male health and wellbeing programs and strengthen the cultural evidence base. One way to progress this would be developing a classification system to help achieve empirical coherence and consistency in the literature. This would allow for a fully powered systematic review of Aboriginal and Torres Strait Islander male health and wellbeing program effectiveness within homogenous groups. In addition, the development of an empirical taxonomy could assist researchers with classification and comparative efforts.^89^ This could include co‐design of outcome measures for indicators of health and wellbeing by Aboriginal and Torres Strait Islander men that are deemed ‘common enough’ as to hold utility for program evaluations. This taxonomy would provide an agreed language that would serve two purposes: (1) to be used as a resource for services to drive health program development, delivery and evaluation within their service and (2) to advance the field of Aboriginal and Torres Strait Islander male health by providing best practice and evidence‐based elements of an Aboriginal and Torres Strait Islander male health and wellbeing program for delivery in communities nationally.

This scoping review explored and described the evidence base and did not include a critical appraisal of included articles, which limits the influence of findings on policy and practice. Nevertheless, the descriptive findings regarding the programs and their key elements can assist health promotion and primary care practitioners in establishing local health initiatives for Aboriginal and Torres Strait Islander males. Descriptive findings outline strategies to increase Aboriginal and Torres Strait Islander male involvement in health care planning and delivery; and can inform strategies for improving health equity among this cohort. These aspects align with core principles of national, government endorsed strategies. For example, the National Men's Health Strategy 2020–2030^90^ calls for action on; considering the needs and preferences of men in the design, delivery, promotion and continuous improvement of programs and services; and a focus on implementation, monitoring, evaluation and continuous improvement to track progress and optimise outcomes. Furthermore, the National Cultural Respect Framework for Aboriginal and Torres Strait Islander Health 2016–2026^91^ and the National Strategic Framework for Aboriginal and Torres Strait Islander Peoples' Mental Health and Social and Emotional Wellbeing, both set principles for Aboriginal and Torres Strait Islander leadership and partnership,^26^, ^91^ while the latter also provides ‘new approaches’ in delivering health programs for Aboriginal and Torres Strait Islander people that include using a SEWB framework ensuring social and cultural determinants and cultural safety are considered.^26^

## 5 CONCLUSIONS

The size and scope of the body of literature regarding Aboriginal and Torres Strait Islander male health and wellbeing programs needs to be enhanced. Strategies in the identified programs highlighted the strengths of Aboriginal and Torres Strait Islander males, which can inform the greater conceptual and empirical development of male health programs. Further, future program research and evaluation should report information consistently across the seven key elements identified and described in this review. Finally, ongoing, and sustainable investment in Aboriginal and Torres Strait Islander male health and wellbeing can potentially improve the availability and quality of preventative health care for these males.

## FUNDING INFORMATION

KC is supported by an Investigator Grant (Emerging Leadership 1) from the National Health and Medical Research Council (APP1175214) and a Department of Health and Aged Care, Australian Government Grant (MRF206564) through the 2020 Primary Health Care Research Grant Opportunity.

## CONFLICT OF INTEREST STATEMENT

The authors have no conflicts of interest to declare.

## Data Availability

Data sharing is not applicable to this article as no new data were created or analyzed in this study.

## APPENDIX A SEARCH STRATEGY

### A.1 SEARCH STRATEGY (SCHOLARLY LITERATURE AND GREY LITERATURE)

The scholarly literature databases searched were: OvidMedline, Informit, WebOfScience, Scopus, Psychinfo, Proquest, and CINAHL. Below is the complete search strategy for a major database which was adapted for use in the remaining databases.

Complete search strategy for **Ovid MEDLINE(R) and Epub Ahead of Print, In‐Process, In‐Data‐Review and Other Non‐Indexed Citations**:

**Table:**

1	Exp Australia/ or Australia$.ti,ab.
2	Oceanic ancestry group/ or (aborigin$ or indigenous or First People or First Nation).ti,ab.
3	1 and 2
4	torres strait$ islander$.ti,ab.
5	3 or 4
6	Men's Health/
7	exp Masculinity/
8	exp male/ not exp. female/
9	(male* or "“men's” or men or man or Father* or Dad* or Boys or Uncle*).ti.
10	or/6–9
11	5 and 10
12	Community‐Institutional Relations/
13	Health Knowledge, Attitudes, Practice/
14	exp Health Promotion/
15	Smoking Prevention/
16	Social Support/
17	community health services/ or community mental health services/ or community networks/ or exp. community participation/ or family planning services/ or health services, indigenous/ or preventive health services/
18	Health services indigenous/
19	(Program* or Intervention or Development or Project or service* or “feasibility study”).tw,kf.
20	Program Evaluation/ or Program Development/
21	Crisis Intervention/ or Internet‐Based Intervention/ or Early Medical Intervention/ or Psychosocial Intervention/
22	Feasibility Studies/
23	(Exercise* or Health or Resilience or “Social and Emotional Well*” or “Mental Health” or Wellbeing or Cultur* or Communit* or Connectedness or Psychological or Psychosocial or Depression or Stress* or Anxiety or “Substance Use” or trauma* or Stress or suicide or Grief or Heal* or Empower* or Alcohol or tobacco or nutrition or diet or “physical activity” or smok* or drug or Gunja or Marijuana or ice or Methamphetamine or addiction or gambl* or “domestic violence” or “lateral violence” or prison or detention or incarcerat* or “health promot*” or sport or crime or weight).tw,kf
24	or/12–23
25	11 and 24

### A.2 GREY LITERATURE SEARCH

We searched eight key grey literature databases, the strategies were:

**Table:**

Online database	Search terms
Australian Indigenous Health InfoNet	*Contains some*: men OR male OR dad OR uncle OR father OR boys AND program *[title and description]*. AND (2nd search): Population Group ‘Men’ and ‘programs’
Lowitja Institute	Men's Health/all types.
Google	Aboriginal and Torres Strait Islander Men's health programs Aboriginal men's group evaluation report
Healthy Male	‘Aboriginal and Torres Strait’
Australian Men's Sheds Association	‘Aboriginal and Torres Strait’
Australian Men's Health Forum	‘Aboriginal and Torres Strait Islander men’
Beyond Blue	‘Aboriginal and Torres Strait Islander men’
Movember Foundation	‘Aboriginal and Torres Strait Islander’
